# Impact of alcohol consumption on atherosclerosis: a systematic review and meta-analysis

**DOI:** 10.3389/fnut.2025.1563759

**Published:** 2025-04-30

**Authors:** Jiayuan Song, Jiuhua She, Jinzhu Yin, Sihan Hu, Guijun Shi, Liping Chang

**Affiliations:** ^1^College of Integrative Medicine, Changchun University of Chinese Medicine, Changchun, China; ^2^College of Traditional Chinese Medicine, Changchun University of Chinese Medicine, Changchun, China; ^3^Department of Cardiovascular Medicine, Affiliated Hospital of Changchun University of Chinese Medicine, Changchun, China; ^4^School of Public Health, Guangxi Medical University, Guangxi, China; ^5^Department of Cardiovascular Medicine, Changchun Hospital of Traditional Chinese Medicine, Changchun, China

**Keywords:** alcohol, consumption, atherosclerosis, meta-analysis, systematic review

## Abstract

**Introduction:**

Atherosclerosis, a chronic vascular disease, impacts various arterial systems, such as the coronary, carotid, cerebral, renal, and peripheral arteries. Dietary factors, especially alcohol consumption, significantly contribute to the progression of atherosclerosis. However, systematic evaluations of alcohol’s impact on atherosclerosis are still limited. This study investigates the impact of alcohol consumption on atherosclerosis via meta-analysis and assesses the moderating effects of drinking frequency, gender, and other factors.

**Methods:**

By December 2024, a comprehensive literature search was conducted across PubMed, Embase, Cochrane, and Web of Science databases. Studies evaluating the relationship between alcohol consumption and atherosclerosis were rigorously selected and assessed for quality. The study protocol was registered with the INPLASY database. Data extraction and statistical analysis were conducted using STATA 18.0 software. A total of 26 studies involving 326,513 patients across 10 countries were included. Considering that different biological mechanisms may regulate atherosclerosis in different arterial locations, we conducted subgroup analyses to explore differences in country, study type, arterial site, diagnostic criteria, type of alcohol, and gender.

**Result:**

The results show that the overall analysis did not show a significant promoting effect of alcohol consumption on the development of atherosclerosis (OR = 0.91, 95% CI 0.79–1.05). Subgroup analyses revealed several important trends. Alcohol consumption may increase the risk of atherosclerosis in specific countries (Japan, South Korea, Brazil, and Denmark), types of studies (cohort and case–control studies), arterial locations (coronary arteries), and diagnostic criteria (clinical diagnosis and computed tomography). Interestingly, we found that alcohol consumption may increase the risk of atherosclerosis in women. Furthermore, varying levels of alcohol consumption appear to result in differing risks of the disease.

**Conclusion:**

The impact of alcohol consumption on atherosclerosis is not singular and may interact with multiple factors, including environmental factors, lesion location, and individual characteristics.

**Systematic review registration:**

https://inplasy.com/inplasy-2025-1-0031/, INPLASY202510031.

## 1 Introduction

Atherosclerosis is a chronic vascular condition characterized by lipid deposition in the arterial intima, smooth muscle cell proliferation, and increased fibrous tissue, leading to plaque formation, arterial narrowing, hardening, and blood flow obstruction (1). This disease can affect multiple arterial systems throughout the body, including the coronary, carotid, cerebral, renal, and peripheral arteries (2–4). Coronary artery atherosclerosis is the primary cause of coronary heart disease, while atherosclerosis of the carotid and cerebral arteries is strongly linked to stroke (5, 6). Various risk factors contribute to the development of atherosclerosis, including hypertension, hyperlipidemia, diabetes, smoking, obesity, physical inactivity, and genetic predisposition. These factors promote inflammation, damage the endothelium, and accelerate lipid deposition, which in turn drives the progression of the disease (7, 8).

Dietary factors, particularly alcohol consumption, play a significant role in the onset and progression of atherosclerosis (9). The impact of alcohol on this disease, however, remains contentious. Some studies suggest that moderate alcohol intake may slow atherosclerosis progression by increasing high-density lipoprotein cholesterol (HDL-C) levels and exerting antioxidant effects (10, 11). In contrast, excessive alcohol consumption has harmful effects on cardiovascular health, including raising blood pressure, disrupting lipid metabolism, and worsening liver damage, all of which accelerate atherosclerosis development (12). Furthermore, the effects of different types of alcoholic beverages on arterial health can vary. Research indicates that polyphenols found in red wine may offer potent antioxidant benefits (13), suggesting that moderate red wine consumption could potentially help prevent cardiovascular diseases.

This study investigates the relationship between alcohol consumption and the risk of atherosclerosis through a systematic review and meta-analysis. It specifically examines the type of alcoholic beverage, the various sites of atherosclerosis (including the coronary, carotid, and peripheral arteries), and regional differences across countries. The results aim to provide evidence-based guidance for the clinical management of atherosclerosis, supporting the development of precision medicine and personalized treatment strategies.

## 2 Materials and methods

### 2.1 Protocol and registration

To ensure methodological transparency and rigor, this meta-analysis followed the Meta-analysis of Observational Studies in Epidemiology (MOOSE) guidelines and the Preferred Reporting Items for Systematic Reviews and Meta-Analyses (PRISMA) statement (14, 15). These guidelines provide a standardized framework for conducting and reporting systematic reviews and meta-analyses. Additionally, the study protocol has been registered with the International Platform of Registered Systematic Review and Meta-analysis Protocols (INPLASY) database (registration number: INPLASY202510031), further enhancing transparency and minimizing the risk of bias.

### 2.2 Literature search strategy

We conducted a search across four major databases: PubMed, Embase, Cochrane, and Web of Science to ensure comprehensive coverage of relevant literature. The search focused on studies examining the relationship between alcohol consumption and atherosclerosis, spanning from the inception of each database through December 2024. The search terms included: “Alcohol Drinking,” “Ethanol,” “Alcohol Consumption,” “Alcohol Intake,” “Beer,” “Wine,” “Liquor,” “Atherosclerosis,” “Atherogenesis,” “Atherogeneses,” “Limb Atherosclerosis,” “Coronary Atherosclerosis,” “Carotid Atherosclerosis,” “Intracranial Arteriosclerosis,” and “Cerebral Arteriosclerosis.” This systematic search strategy was designed to capture a broad range of relevant studies, thereby enhancing the reliability and comprehensiveness of the findings. The detailed search strategies are provided in Supplementary Tables 1–4.

### 2.3 Inclusion criteria

To evaluate the relationship between alcohol consumption and atherosclerosis, only studies involving patients diagnosed with atherosclerosis were included. There were no restrictions on study type; however, studies were required to report multivariable-adjusted statistical measures, such as relative risk (RR), odds ratio (OR), hazard ratio (HR), and 95% confidence intervals (CI). To maintain scientific rigor, only cohort, case–control, and cross-sectional studies were deemed eligible for inclusion in the analysis.

### 2.4 Exclusion criteria

The following studies were excluded from the analysis: studies that did not involve patients diagnosed with atherosclerosis; Studies whose results did not meet the inclusion criteria; reviews, case studies, survey analyses, conference abstracts, and irrelevant literature; duplicate publications.

### 2.5 Literature screening and data extraction

Based on predefined criteria, two reviewers (Song and Hu) independently screened the titles, abstracts, and full texts of articles retrieved from various databases to assess their eligibility for inclusion. In case of disagreement, the original articles were re-examined, and consensus was reached through discussion. Relevant data were extracted from the selected studies, including author names, publication year, country, study type, alcohol type, atherosclerosis site, diagnostic criteria, sample size, age, gender, odds ratio, lower confidence interval, upper confidence interval, grouping basis and covariate adjustments. This data was then organized into a Table 1 for systematic analysis.

**Table 1:** Characteristics of included studies.

Inclusion in the study	Country	OR	LCI	UCI	Type of study	Type of alcohol	Atherosclerotic site	Diagnostic criteria	Sample size	Age	Gender (male/female)	Grouping basis	Adjustments
Adeleye Dorcas Omisore 2018 (21)	Nigeria	2.2	0.92	5.25	Cross-sectional studies	/	Carotid artery	Ultrasound	162	>18	80/82		Age (years), Gender, Smoking, Hypertension, Diabetes, Dyslipidaemia, Alcohol, CKD, Obesity
Akihiko Krtamur 1998 (22)	Japan	0.59	0.23	1.51	Cross-sectional studies	/	Coronary artery	Clinical diagnosis	8,476	40–59	8,476/0		Age, serum total cholesterol level, blood pressure, body mass index, cigarette smoking, history of diabetes mellitus, and electrocardiographic evidence of left ventricular hypertrophy
Annie Britton 2004 (23)	Britain	1.23	0.83	1.83	Cohort studies	Beer	Coronary artery	Clinical diagnosis	10,308	35–55	6,895/3,413	male	Age, smoking (no/ex/light/moderate/heavy), employment grade (high/medium/low), blood cholesterol, blood pressure, body mass index, general health questionnaire score.
		0.81	0.33	2.01								female	
Belén Moreno-Franco 2020 (24)	Spain	0.69	0.21	2.13	Cross-sectional studies	/	Femoral artery	Ultrasound	2,099	39–59	2,099/0	Never-smokers	age, hypertension, dyslipidaemia and diabetes
		4.39	2.16	8.93								Ever-smokers	
Chun Zhang 2022 (25)	China	0.749	0.588	0.953	Cross-sectional studies	/	Carotid artery	Ultrasound	47,063	≥18	27,766/19,297	no fatty liver disease	sex, age, BMI, Smoking, Alcohol, Physical activity, Sedentary behavior, Dietary diversity, Carotid artery Disease
		0.829	0.652	1.056								fatty liver disease	
Dong Hyun Sinn 2014 (26)	South Korea	0.74	0.6	0.92	Cross-sectional studies	/	Carotid artery	Ultrasound	2,280	≥30	2,280/0		Age, BMI, Current smoking, Metabolic syndrome
Dwayne Reed 1991 (27)	Japan	0.64	0.44	0.87	Cohort studies	/	Coronary artery	Clinical diagnosis	7,591	45–68	7,591/0		Age, Systolic blood pressure, Serum cholesterol, BMI, Alcohol intake, Serum glucose, Serum triglyceride, Cigarette smoking, Physical activity index, Cholesterol intake per 1,000 calories
Flávio D.Fuchs 2004	Brazil	0.69	0.40	1.18	Cohort studies	Beer wine liquor	Coronary artery	Clinical diagnosis	14,506	54.6 ± 5.7	6,276/8,230	male; white	Mean age, Highest level of education, Annual income, Mean body mass index, Body mass index category, Mean blood pressure, Current drinker, Rare drinker, Current smoker, Parental history of hypertension, Poor self-perceived health, Diabetes mellitus
		0.55	0.27	1.13						53.9 ± 5.7		female; white	
		1.10	0.40	2.98						53.7 ± 6.0		male; black	
		0.49	0.20	1.18						53.3 ± 5.7		female; black	
Franziska K Bishop 2009 (29)	America	0.9	0.8	1.1	Cross-sectional studies	/	Coronary artery	Clinical diagnosis	1,306	20–60	637/669		Age, Race/ethnicity, Education, Income, BMI, Visceral fat, Average Waist, Waist to Hip, Cholesterol, Smoking, Physical Activity, Alcohol drinks
Hermann Brenner 2001 (30)	Germany	0.76	0.48	1.20	Case–control studies	Beer wine	Coronary artery	Coronary angiography	791	40–68	626/165		Age, Gender, Education, Married, Smoking status, Body mass index, History of diabetes mellitus
Janne Tolstrup 2006 (31)	Denmark	0.59	0.48	0.71	Cohort studies	/	Coronary artery	Clinical diagnosis	53,500	50–65	25,052/28,448	male	Age, Alcohol intake, Smoking, level of education, Physical activity, Body mass index, Vegetable intake, Fruit intake, Fish intake, Saturated fat
		0.65	0.51	0.84								female	
Jeanne K.Tofferi 2004 (32)	America	1.25	0.76	2.07	Cohort studies	Beer Wine liquor	Coronary artery	Computed tomography	725	39–45	602/123		Age, Gender, White, College educated, Cardiac risk factors, 5-Y Framingham risk index, Percent of daily drinkers, Coronary artery calcification score
Jurgen T.Rehm 1997 (33)	America	2.5556	1.1912	5.483	Cohort studies	/	Coronary artery	Clinical diagnosis	6,788	58.1 ± 10.3	2,960/3,828	male	Age, Body mass index, drink, smoke
		0.6199	0.3598	1.0682						57 ± 10.9		female	
Koichi Handa 1990 (34)	Japan	1.12	0.48	2.63	Cohort studies	/	Coronary artery	Coronary angiography	212	/	212/0		Age, Total cholesterol, Triglyceride, HDL cholesterol, Cigarette smoking, Hypertension
Marcello Ricardo Paulista Markus 2015 (35)	Germany	1.56	0.96	2.51	Cross-sectional studies	/	Aorta	Clinical diagnosis	2,022	45–81	1,035/987		Age, Gender, Years of education, Smoking, Physical inactivity, Height, Body mass index, Waist circumference, Waist-to-height ratio, Systolic blood pressure, Hypertension, Antihypertensive Medication, Glycohemoglobin
Mark J.Pletcher 2005 (36)	America	1.5	0.9	2.7	Cross-sectional studies	/	Coronary artery	Computed tomography	3,037	33–45	1,379/1,658		Age, Gender, Educational level, Annual income, Cigarette smoking, Self-rated physical activity level, Body mass index, Family history of premature coronary heart disease
Michiko Fujisawa 2008 (37)	Japan	0.22	0.06	0.8	Cross-sectional studies	/	Carotid artery	Ultrasound	136	84.6 ± 0.5	53/83	male	Age, Body mass index, Systolic blood pressure, Diastolic blood pressure, Orthostatic change of blood Pressure, Blood chemical findings, max IMT
		0.17	0.02	1.32						82.9 ± 0.4		female	
Mihaela Tanasescu 2001 (38)	America	0.48	0.27	0.86	Cohort studies	/	Coronary artery	Clinical diagnosis	2,419	40–75	2,419/0	liquor	Age, physical activity, body mass index, smoking, history of hypertension, high cholesterol, family history of MI, use of vitamin E supplements and dietary intake of trans fat, polyunsaturated fat, fiber, folate and total calories.
		0.48	0.20	1.14								Beer	
		0.75	0.37	1.52								wine	
Qi Cheng 2022 (39)	China	0.888	0.586	1.344	Cross-sectional studies	/	Carotid artery	Ultrasound	7,908	57.75 ± 9.45	3,044/4,864		Age, Gender, Smoking, Alcohol drinking, BMI, Waist circumference, Overweight, Obesity, Hemodynamics, Heart rate, Lipids and glucose
Tianyu Zhou 2023 (40)	China	1.42	1.14	1.76	Cohort studies	/	Carotid artery	Ultrasound	22,384	30–79	8,503/13,881		Age, Study area, Education level, Household income, Smoking status, Systolic blood pressure, Body mass index, Low-density lipoprotein cholesterol
Umed A.Ajani 2015 (41)	America	0.67	0.57	0.78	Case–control studies	/	Coronary artery	Clinical diagnosis	87,938	55.4 ± 10.8/52.8 ± 10.1/53.1 ± 9.7/57.3 ± 10.3/63.1 ± 10.0/60.1 ± 10.2/61.4 ± 9.8/63.8 ± 9.1	87,938/0		Age, Smoking, Exercise ≥ 1/wk., Angina, Body mass index, Hypertension, High cholesterol, Aspirin intake
William B.Kannel 1996 (42)	America	0.4	0.2	0.8	Cohort studies	/	Coronary artery	Clinical diagnosis	148	/	81/67		/
Xiaohuan Chen 2024 (43)	China	1.746	0.831	3.772	Cross-sectional studies	/	Carotid artery	Ultrasound	435	≥18	291/144		Age, Gender, Course of disease, BMI, Hypertension, Cerebral infarction, Coronary heart disease, The history of smoking, The history of drinking, Total bilirubin, Blood urea nitrogen
Yann Le Strat 2011 (44)	America	1.14	0.99	1.3	Cross-sectional studies	/	Coronary artery	Clinical diagnosis	41,763	≥18	17,895/23,868		Age, Gender, Race/ethnicity, Educational level, Marital status, Income, Urbanicity, Region, BMI, Alcohol dependence prior to the last 12 months, Lifetime DSM-IV nicotine dependence
Yinze Ji 2024 (45)	China	1.34	1.06	1.71	Cross-sectional studies	/	Coronary artery	Computed tomography	1,528	≥18	1,109/419		Age, Gender, Hypertension, Diabetes, Smoking history, Alcohol consumption history, Aspirin, Statins, eGFR, apoA1
Yiti Liu 2024 (46)	China	2.71	1.36	5.41	Cross-sectional studies	/	Carotid artery	Computed tomography	988	≥ 45	687/301		Age, Gender, BMI, Hypertension, Diabetes, Coronary heart disease, Gastrointestinal bleeding, Pneumonia, Smoking status

### 2.6 Literature screening and data extraction

Two assessors evaluated the quality of the studies using criteria recommended by the Agency for Healthcare Research and Quality (AHRQ) (16). The following aspects were assessed: Whether the data source was clearly defined; Whether the inclusion and exclusion criteria for both exposed and non-exposed groups were provided or referenced from previous studies; Whether the time frame for identifying patients was specified; If not population-based, whether study subjects were selected consecutively; Whether the assessors’ subjective biases influenced other aspects of the study subjects; Whether quality assurance measures were described; Whether the reasons for excluding patients from the analysis were explained; Whether measures to evaluate or control for confounding factors were provided; If applicable, whether the handling of missing data was explained; Whether the response rate and data collection completeness were summarized; If follow-up occurred, whether the percentage of incomplete patient data or follow-up results were identified; Each of these 11 items was evaluated using responses of “yes,” “no,” or “unclear.” We have provided the MOOSE and PRISMA checklists for this paper in the Supplementary file.

### 2.7 Statistical analysis

We conducted a meta-analysis using STATA 18.0 software, following these steps: First, we selected the odds ratio (OR) as the effect size indicator and calculated its 95% confidence interval (CI) to assess statistical significance. Next, we performed heterogeneity testing, using *p* values and I^2^ to guide our analysis. If *p* > 0.1 and I^2^ ≤ 50%, we concluded that heterogeneity was low and applied the fixed-effect model (FE). Conversely, if *p* ≤ 0.1 and I^2^ > 50%, we considered the heterogeneity high and used the random-effect model (RE) (17). To assess publication bias, we generated funnel plots and conducted Egger’s test, examining potential biases in studies on alcohol consumption and atherosclerosis (18). We also performed sensitivity analysis by excluding specific studies to test the robustness of our results (19). Additionally, we conducted a meta-subgroup analysis to investigate the sources of heterogeneity and evaluate its impact on the study outcomes (20).

## 3 Results

### 3.1 Results of literature search

**Figure 1:**
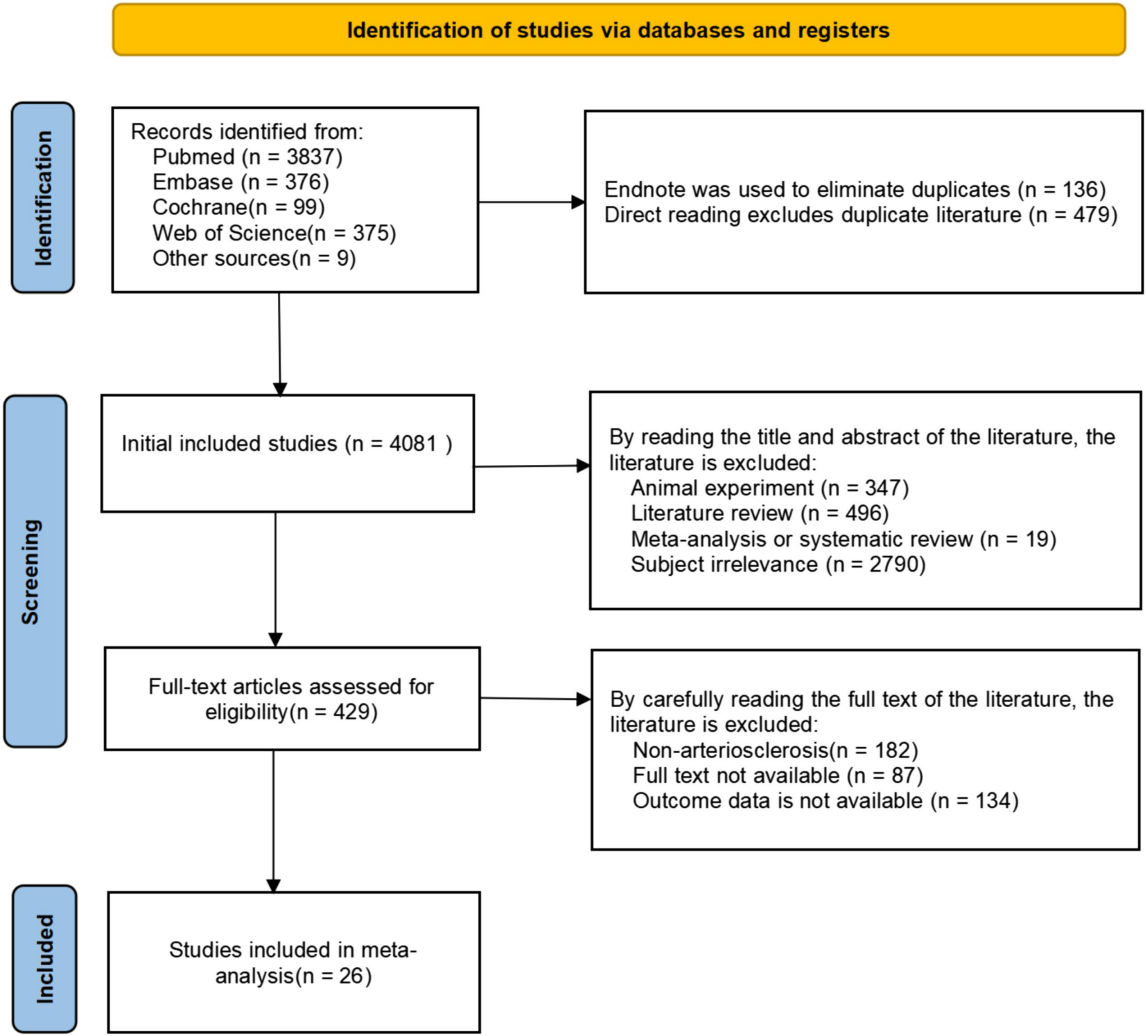
Flowchart of literature selection.

Through a review of other published studies on alcohol consumption and atherosclerosis, we included 9 additional articles. Initially, 4,696 articles were retrieved, of which 4,081 remained after initial included studies. We then carefully assessed the titles, abstracts, and full texts of these articles based on predefined inclusion and exclusion criteria, including study type, participant characteristics, and interventions. As a result, 26 studies were ultimately included (Figure 1), providing a solid data foundation for the subsequent meta-analysis.

### 3.2 Results of included study characteristics

Between 1990 and 2024, 26 studies involving a total of 326,513 patients were conducted across 10 countries: Nigeria, Japan, Britain, Spain, China, South Korea, Brazil, America, Germany, and Denmark (21–46). These studies included 2 case–control studies, 10 cohort studies, and 14 cross-sectional studies. Alcohol type was reported in four studies, which primarily focused on beer, liquor, and wine. Atherosclerosis was studied in different arterial sites: 1 study on femoral artery atherosclerosis, 1 on aortic atherosclerosis, 16 on coronary artery atherosclerosis, and 8 on carotid artery atherosclerosis. The patient age range was broad, with all participants being over 18 years old. There were 215,986 male and 110,527 female patients (Table 1). The AHRQ quality evaluation of the 26 studies is presented as follows (Supplementary Table 5).

### 3.3 Results of meta-analysis

#### 3.3.1 Relationship between alcohol consumption and atherosclerosis

Twenty-six studies have examined the relationship between alcohol consumption and atherosclerosis. The heterogeneity test (*p <* 0.001, I^2^ = 78.8%) revealed significant variability, prompting the use of a random-effects model for effect size synthesis. The meta-analysis found no significant association between alcohol consumption and atherosclerosis (OR = 0.91, 95% CI 0.79–1.05; Supplementary Figure 1). The funnel plot displayed slightly more studies on the left side than the right, with some studies outside the 95% confidence interval. The funnel plot displays the relationship between the effect size (OR) of each study and its standard error (SE). The asymmetric distribution of the points, especially some on the right side located outside the pseudo 95% confidence intervals of the funnel plot, may indicate the presence of publication bias. Egger’s Test (*p* = 0.874, > 0.05) shows the relationship between the precision of each study and the standardized normal deviation (SND) of the effect estimate. The regression line indicates a negative correlation between the effect estimate and precision, which may suggest the presence of a small-study effect. The 95% confidence interval (CI) in the plot is used to assess the uncertainty of the intercept (Supplementary Figures 2–3). Sensitivity analysis confirmed that the results remained consistent even after sequentially excluding each study, demonstrating the robustness of the findings (Supplementary Figure 4). Overall, these results are considered reliable.

While no overall association between alcohol consumption and atherosclerosis was found, the observed heterogeneity, along with the inclusion of studies on atherosclerosis from various arterial locations, may have introduced bias. To address this, we conducted subgroup analyses based on country, study type, arterial location, diagnostic criteria, alcohol type, and gender. Additionally, we examined the relationship between alcohol consumption dose/frequency and atherosclerosis to explore potential influencing factors and assess alcohol’s effects under specific conditions. These subgroup analyses provide a deeper understanding of the sources of heterogeneity and offer more targeted insights for future research, ultimately contributing more precise evidence for clinical practice and public health policy development.

#### 3.3.2 Subgroup analysis—country

**Figure 2:**
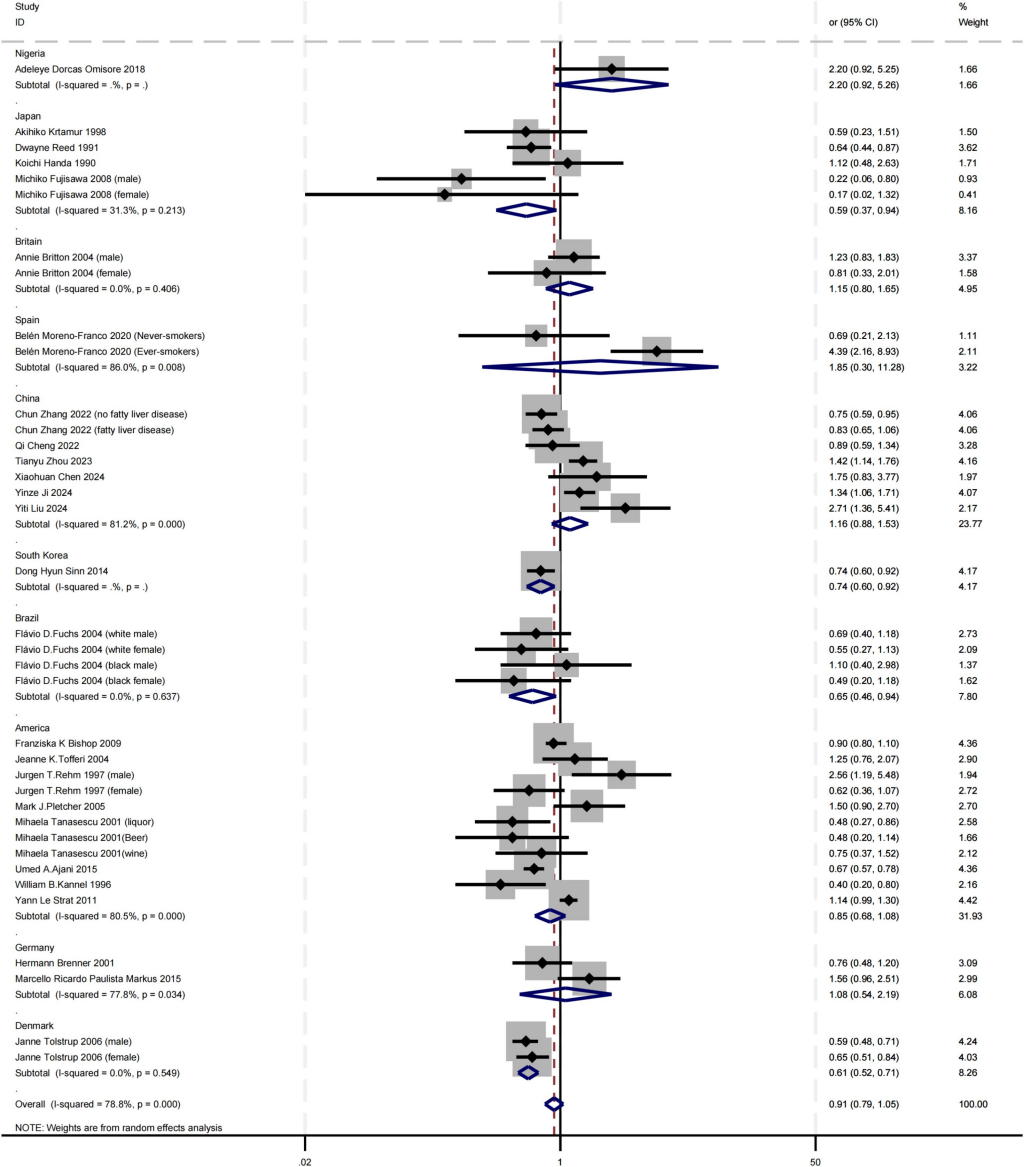
Subgroup analysis of the relationship between alcohol consumption and atherosclerosis—forest plot by country.

Subgroup analyses were conducted across various countries. Among the included studies, heterogeneity was higher in China (I^2^ = 81.2%), Germany (I^2^ = 77.8%), Spain (I^2^ = 86.0%), and America (I^2^ = 80.5%). In contrast, heterogeneity was lower in Japan (I^2^ = 31.3%), Britain (I^2^ = 0.0%), Brazil (I^2^ = 0.0%), and Denmark (I^2^ = 0.0%), leading to the application of a random-effects model. Heterogeneity could not be calculated for countries with only a single study, such as Nigeria and South Korea, due to insufficient data. The findings indicated that alcohol consumption significantly increased the risk of atherosclerosis in Japan (OR = 0.59, 95% CI 0.37–0.94), South Korea (OR = 0.74, 95% CI 0.60–0.92), Brazil (OR = 0.65, 95% CI 0.46–0.94) and Denmark (OR = 0.61, 95% CI 0.52–0.71). In contrast, no significant association was observed in other countries with single studies, including Nigeria, Britain, Spain. These findings suggest that the relationship between alcohol consumption and atherosclerosis may not be solely determined by alcohol intake but is also influenced by region-specific cultural, environmental, and genetic factors (Figure 2).

#### 3.3.3 Subgroup analysis—study type

**Figure 3:**
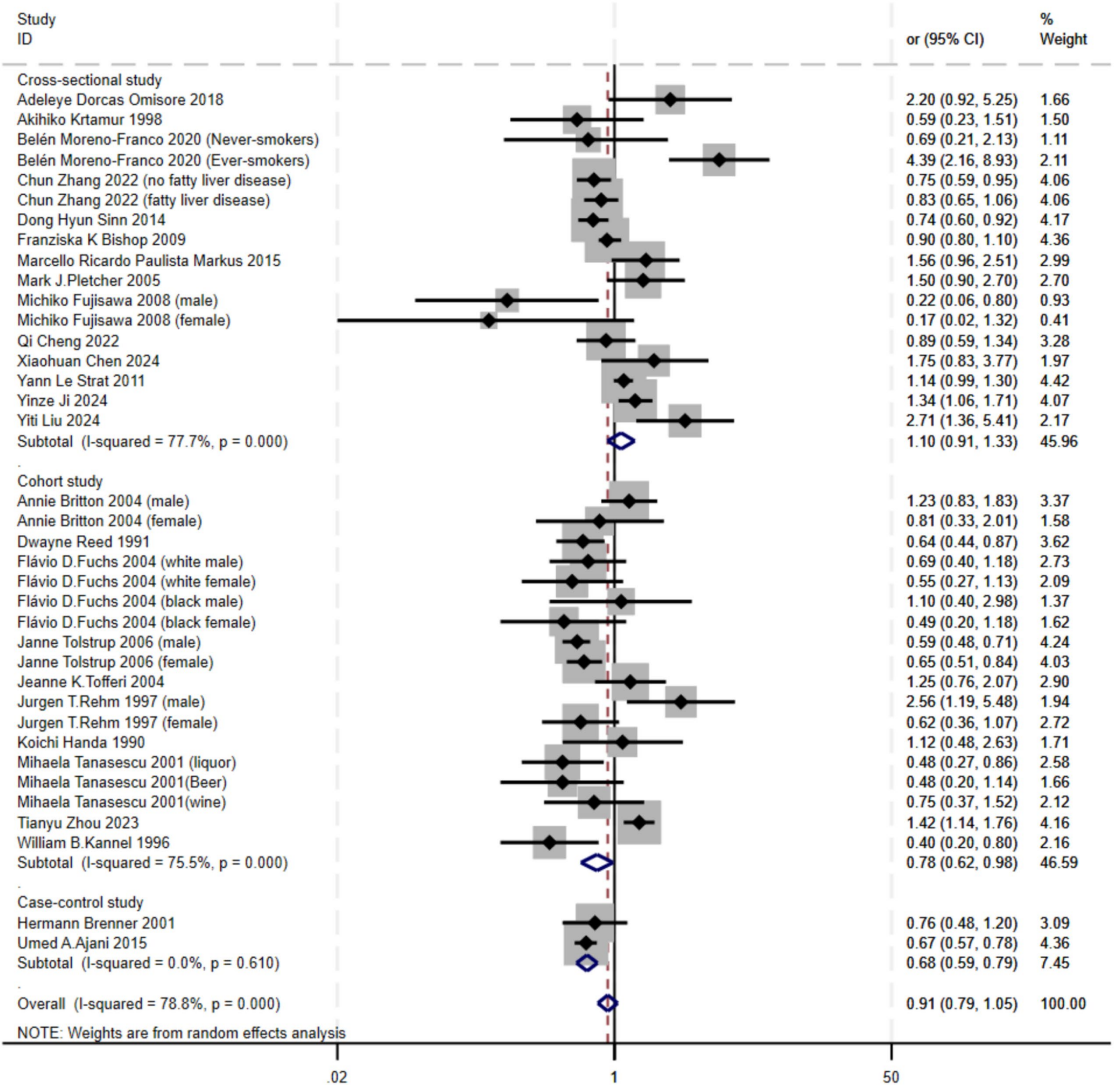
Subgroup analysis of the relationship between alcohol consumption and atherosclerosis—forest plot by study type.

Subgroup analyses were conducted based on study type, revealing varying levels of heterogeneity. High heterogeneity was observed in Cross-sectional studies (I^2^ = 77.7%) and Cohort studies (I^2^ = 75.5%), while Case–control studies showed no heterogeneity (I^2^ = 0.0%). As a result, a random-effects model was applied. The findings indicated that alcohol consumption significantly increased the risk of atherosclerosis in both Cohort studies (OR = 0.78, 95% CI 0.62–0.98) and Case–control studies (OR = 0.68, 95% CI 0.59–0.79). In contrast, no significant relationship was found in Cross-sectional studies. This discrepancy may be due to the ability of Cohort and Case–control studies to better capture causal relationships, which Cross-sectional studies may not adequately reflect (Figure 3).

#### 3.3.4 Subgroup analysis—atherosclerotic sites

**Figure 4:**
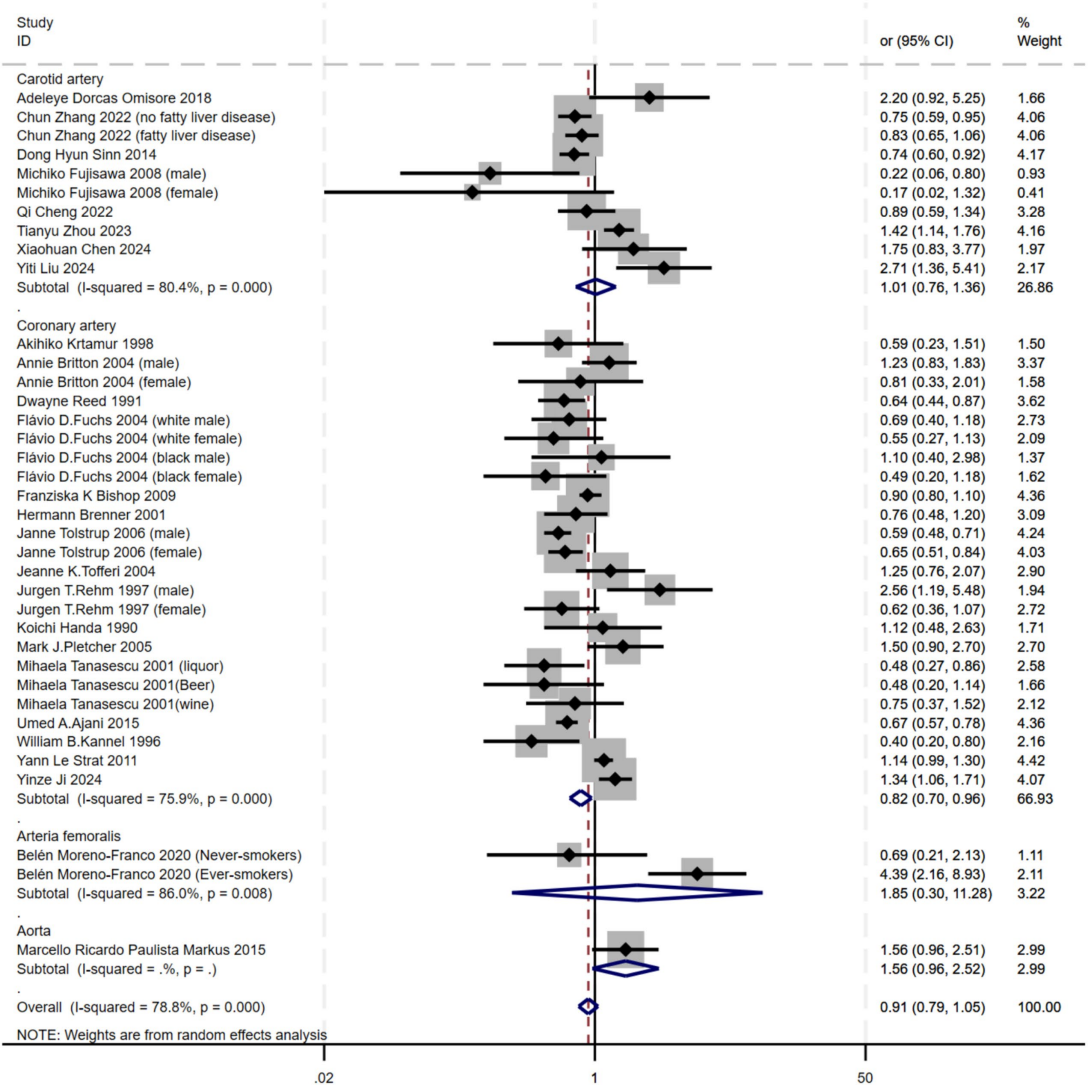
Subgroup analysis of the relationship between alcohol consumption and atherosclerosis—forest plot by artery location.

Subgroup analyses were conducted for different arterial locations. Among the included studies, only the Carotid artery (I^2^ = 80.4%), Femoral artery (I^2^ = 86.0%) and Coronary artery (I^2^ = 75.9%) had more than one study, allowing for heterogeneity assessment. High heterogeneity was observed in these arterial locations, prompting the use of a random-effects model. For the Aorta only a single study, heterogeneity could not be calculated due to insufficient data. The findings indicated that alcohol consumption increased the risk of disease in the Coronary artery (OR = 0.82, 95% CI 0.70–0.96). However, in the Carotid artery, Femoral artery and Aorta, the effect of alcohol was either weak or appeared to reduce the risk of atherosclerosis. This suggests that alcohol’s impact may vary across different arterial locations. Such differences could be attributed to factors like genetics, lifestyle, or limitations in sample size and study design, which may prevent drawing consistent conclusions (Figure 4).

#### 3.3.5 Subgroup analysis—diagnostic criteria

**Figure 5:**
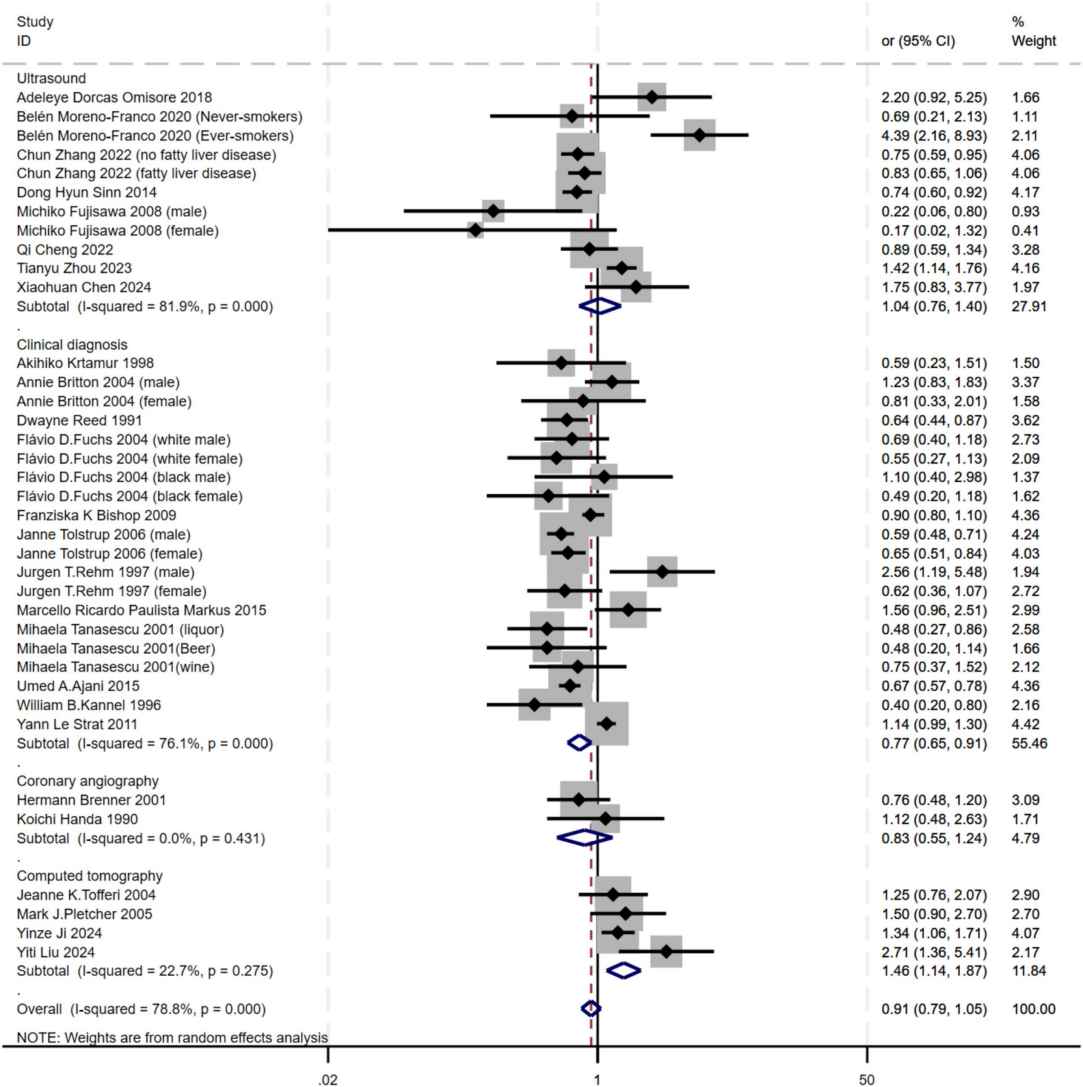
Subgroup analysis of the relationship between alcohol consumption and atherosclerosis—forest plot by diagnostic criteria.

Subgroup analyses were performed based on diagnostic criteria. Ultrasound (I^2^ = 81.9%) and Clinical diagnosis (I^2^ = 76.1%) exhibited high heterogeneity, while Computed tomography (I^2^ = 22.7%) showed moderate heterogeneity, and Coronary angiography (I^2^ = 0.0%) showed no heterogeneity. As a result, a random-effects model was applied. The findings indicated a significant association between alcohol consumption and atherosclerosis in both Clinical diagnosis (OR = 0.77, 95% CI 0.65–0.91) and Computed tomography (OR = 1.46, 95% CI 1.14–1.87). However, no such association was observed with Ultrasound or Coronary angiography. These differences may be related to variations in how atherosclerosis is diagnosed across different arterial locations (Figure 5).

#### 3.3.6 Subgroup analysis—gender

**Figure 6:**
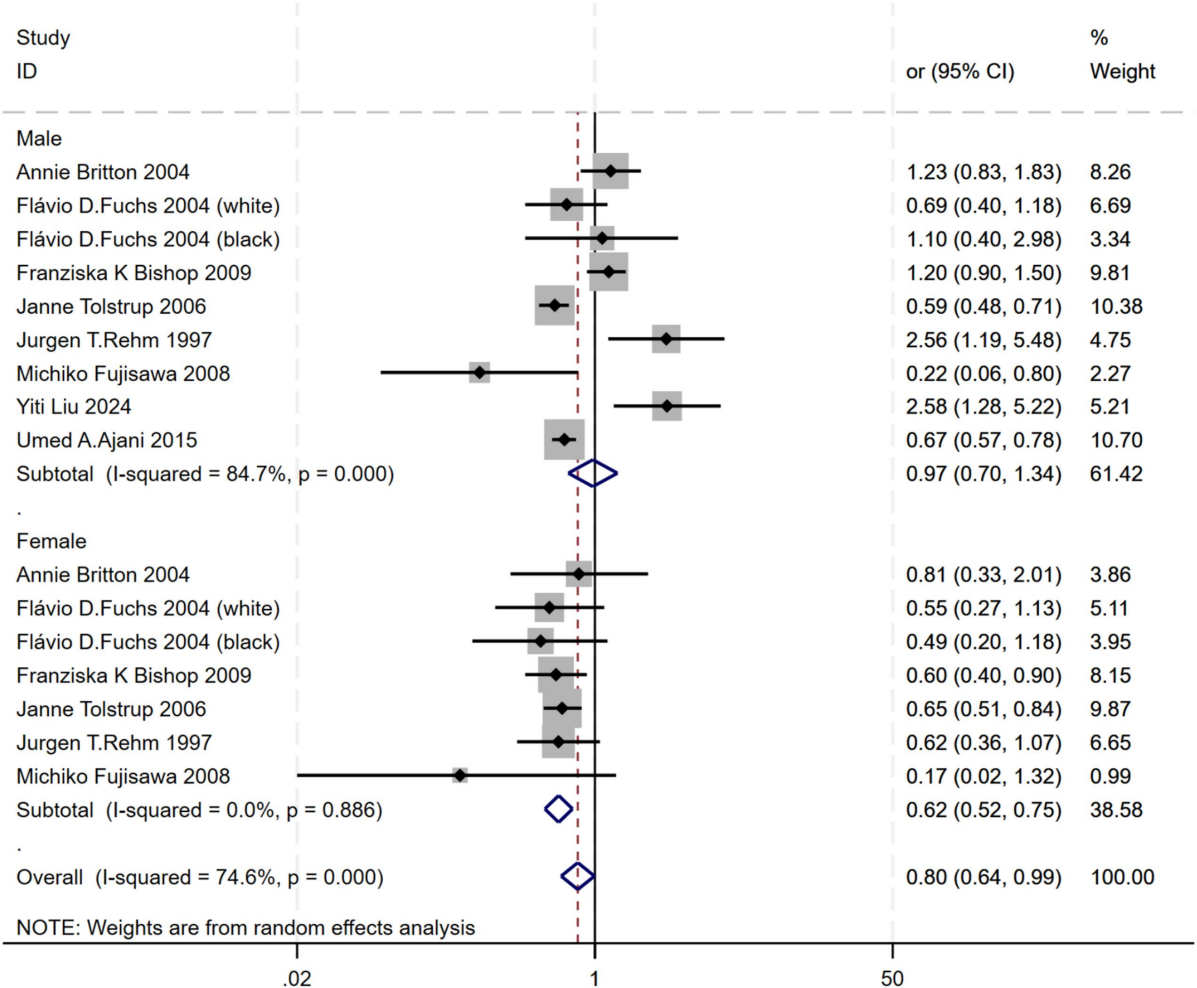
Subgroup analysis of the relationship between alcohol consumption and gender—forest plot by diagnostic criteria.

Subgroup analyses were conducted based on gender. Due to the outlier values for Qi Cheng 2022 (males) OR = 5.693, 95% CI 3.028 to 14.415, and Qi Cheng 2022 (females) OR = 27.737, 95% CI 3.508 to 51.967, these were excluded from the gender analysis. Since males (I^2^ = 84.7%) showed high heterogeneity, while females (I^2^ = 0.0%) did not exhibit heterogeneity, a random-effects model was applied. A significant association between alcohol consumption and atherosclerosis was found in females (OR = 0.62, 95% CI 0.52–0.75). However, no such association was observed in males (OR = 0.97, 95% CI 0.70–1.34). This suggests that the relationship between alcohol consumption and atherosclerosis is influenced by gender (Figure 6).

#### 3.3.7 Subgroup analysis—wine

Subgroup analyses were conducted based on the type of alcohol consumed. Beer (I^2^ = 43.7%) and wine (I^2^ = 0.0%) showed low heterogeneity, while liquor (I^2^ = 74.7%) exhibited high heterogeneity. As a result, a random-effects model was applied. The findings indicated no significant association between alcohol consumption and atherosclerosis for beer [OR = 0.90, 95% CI 0.67–1.19, wine (OR = 1.03, 95% CI 0.81–1.32), or liquor (OR = 1.01, 95% CI 0.60–1.72)]. This suggests that different types of alcohol may not have distinct effects on atherosclerosis risk, and may even reduce the risk in some cases (Supplementary Figure 5).

#### 3.3.8 Alcohol consumption dose/frequency and atherosclerosis

Seventeen studies have reported on the relationship between alcohol consumption dose/frequency and atherosclerosis. For example, Akihiko Kitamura (1998) found a relationship at an alcohol intake of 46–68 g/day; Belén Moreno-Franco (2020, ever-smokers) found a relationship at an alcohol intake of 2-30 g/day. Dong Hyun Sinn (2014) at less than 10 g/day; Dwayne Reed (1991) at 52 mL/day; Janne Tolstrup (2006, male) at 2–4 days/week, 5–6 days/week, and 7 days/week; Janne Tolstrup (2006, female) at 1 day/week, 2–4 days/week, and 7 days/week; Jurgen T. Rehm (1997, male) at less than 2 drinks/week, 2–7 drinks/week, 8–14 drinks/week, 15–28 drinks/week, and 29–42 drinks/week; Jurgen T. Rehm (1997, female) at 2–7 drinks/week; Koichi Handa (1990) at moderate alcohol intake; Umed A. Ajani (2015) at daily alcohol intake; William B. Kannel (1996) at 5.0–14.9 g/day and greater than 25 g/day; and Yann Le Strat (2011) at moderate consumption over 12 months and hazardous drinking over 12 months. Collectively, these studies suggest that alcohol consumption is linked to atherosclerosis, with varying levels of intake potentially contributing to different risks of developing the condition (Table 2).

**Table 2:** Relationship between alcohol consumption dose/frequency and atherosclerosis.

Inclusion in the study	Sample size	OR	95%CI		Alcohol dosage/frequency
			LCI	UCI	
Akihiko Krtamur 1998	2,317	0.69	0.37	1.29	1-22 g/d
Akihiko Krtamur 1998	2,419	0.55	0.29	1.05	23-45 g/d
Akihiko Krtamur 1998*	**1,667**	**0.41**	**0.19**	**0.88**	**46-68 g/d**
Akihiko Krtamur 1998	580	0.59	0.23	1.51	≥69 g/d
Annie Britton 2004(male)	93	1	0.78	1.23	1-2time/month
Annie Britton 2004(male)	219	0.97	0.80	1.12	Daily
Annie Britton 2004(male)	41	1.23	0.83	1.83	≥2time/day
Annie Britton 2004(female)	61	1.15	0.84	1.57	1-2time/month
Annie Britton 2004(female)	59	0.72	0.50	1.02	Daily
Annie Britton 2004(female)	7	0.81	0.33	2.01	≥2time/month
Belén Moreno-Franco 2020(Never-smokers)	104	0.95	0.53	1.72	2-30 g/day
Belén Moreno-Franco 2020(Never-smokers)	33	1.14	0.56	2.32	30-60 g/d
Belén Moreno-Franco 2020(Never-smokers)	6	0.69	0.21	2.13	≥60 g/day
Belén Moreno-Franco 2020(Ever-smokers)*	**550**	**2.59**	**1.49**	**4.48**	**2-30 g/day**
Belén Moreno-Franco 2020(Ever-smokers)	280	3.17	1.79	5.61	30-60 g/d
Belén Moreno-Franco 2020(Ever-smokers)	70	4.39	2.16	8.93	≥60 g/day
Dong Hyun Sinn 2014*	**452**	**0.71**	**0.56**	**0.90**	**<10 g/day**
Dong Hyun Sinn 2014	456	0.80	0.62	1.04	10-20 g/day
Dwayne Reed 1991*	**/**	**0.64**	**0.44**	**0.87**	**52 mL/day**
Flávio D.Fuchs 2004(white male)	1,141	1.05	0.7	1.58	1-70 g/week
Flávio D.Fuchs 2004(white male)	633	0.81	0.5	1.32	70 g-140 g/week
Flávio D.Fuchs 2004(white male)	368	0.81	0.45	1.44	140 g-210 g/week
Flávio D.Fuchs 2004(white male)	503	0.69	0.4	1.18	≥210 g/week
Flávio D.Fuchs 2004(white female)	1,250	0.64	0.36	1.12	1-70 g/week
Flávio D.Fuchs 2004(white female)	703	0.55	0.27	1.13	≥70 g/week
Flávio D.Fuchs 2004(black male)	247	1.80	0.86	3.76	1-70 g/week
Flávio D.Fuchs 2004(black male)	171	1.13	0.45	2.84	70 g-140 g/week
Flávio D.Fuchs 2004(black male)	90	2.67	1.12	6.34	140 g-210 g/week
Flávio D.Fuchs 2004(black male)	147	1.10	0.40	2.98	≥210 g/week
Flávio D.Fuchs 2004(black female)	372	0.49	0.20	1.18	≥1 g/week
Hermann Brenner 2001	102	0.71	0.44	1.15	≤125 g/Week
Hermann Brenner 2001	121	0.84	0.51	1.40	>125 g/Week
Janne Tolstrup 2006(male)	140	0.93	0.75	1.16	1 days/week
Janne Tolstrup 2006(male)*	424	**0.78**	**0.66**	**0.94**	**2–4 days/week**
Janne Tolstrup 2006(male)*	195	**0.71**	**0.57**	**0.87**	**5-6 days/week**
Janne Tolstrup 2006(male)*	305	**0.59**	**0.48**	**0.71**	**7 days/week**
Janne Tolstrup 2006(female)*	95	**0.64**	**0.51**	**0.81**	**1 days/week**
Janne Tolstrup 2006(female)*	187	**0.63**	**0.52**	**0.77**	**2–4 days/week**
Janne Tolstrup 2006(female)	77	0.79	0.61	1.03	5-6 days/week
Janne Tolstrup 2006(female)*	90	**0.65**	**0.51**	**0.84**	**7 days/week**
Jeanne K.Tofferi 2004	313	1.02	0.64	1.63	<1 Drink/day
Jeanne K.Tofferi 2004	95	1.13	0.59	2.15	1–2 Drink/day
Jeanne K.Tofferi 2004	62	1.26	0.69	2.59	>2 Drink/day
Jurgen T.Rehm 1997(male)*	977	**0.7641**	**0.6135**	**0.9518**	**<2 Drink/week**
Jurgen T.Rehm 1997(male)*	665	**0.6205**	**0.4858**	**0.7924**	**2–7 Drink/week**
Jurgen T.Rehm 1997(male)*	272	**0.6461**	**0.4756**	**0.8776**	**8–14 Drink/week**
Jurgen T.Rehm 1997(male)*	217	**0.6632**	**0.4739**	**0.9283**	**15–28 Drink/week**
Jurgen T.Rehm 1997(male)*	89	**0.5135**	**0.2978**	**0.8853**	**29–42 Drink/week**
Jurgen T.Rehm 1997(male)	61	0.6199	0.3598	1.0682	>42 Drink/week
Jurgen T.Rehm 1997(female)	1,661	0.9276	0.7791	1.1044	<2 Drink/week
Jurgen T.Rehm 1997(female)*	507	**0.5068**	**0.3668**	**0.7003**	**2–7 Drink/week**
Jurgen T.Rehm 1997(female)	127	0.6172	0.3508	1.0861	8–14 Drink/week
Jurgen T.Rehm 1997(female)	73	0.6083	0.3103	1.1927	15–28 Drink/week
Jurgen T.Rehm 1997(female)	10	2.5556	1.1912	5.4830	>42 Drink/week
Koichi Handa 1990	21	1.22	0.4	3.71	Light
Koichi Handa 1990*	80	**0.29**	**0.13**	**0.63**	**Moderate**
Koichi Handa 1990	53	1.12	0.48	2.63	Heavy
Marcello Ricardo Paulista Markus 2015	209	1.07	0.94	1.22	20 g/day
Marcello Ricardo Paulista Markus 2015		1.20	0.94	1.53	30 g/day
Marcello Ricardo Paulista Markus 2015	64	1.32	0.94	1.84	40 g/day
Marcello Ricardo Paulista Markus 2015		1.42	0.95	2.11	50 g/day
Marcello Ricardo Paulista Markus 2015	43	1.48	0.96	2.30	60 g/day
Marcello Ricardo Paulista Markus 2015		1.54	0.96	2.45	70 g/day
Marcello Ricardo Paulista Markus 2015		1.56	0.96	2.51	80 g/day
Mark J.Pletcher 2005	933	1.1	0.8	1.5	1–6 Drink/week
Mark J.Pletcher 2005	315	1.3	0.8	2.1	7–13 Drink/week
Mark J.Pletcher 2005	218	1.5	0.9	2.7	≥14 Drink/week
Umed A.Ajani 2015	225	0.93	0.78	1.12	Monthly
Umed A.Ajani 2015	939	0.89	0.78	1.02	weekly
Umed A.Ajani 2015*	436	**0.67**	**0.57**	**0.78**	**Daily**
William B.Kannel 1996	**/**	0.8	0.5	1.2	<1.5 g/day
William B.Kannel 1996	**/**	0.6	0.4	1	1.5–4.9 g/day
William B.Kannel 1996*	**/**	**0.6**	**0.4**	**0.9**	**5.0–14.9 g/day**
William B.Kannel 1996	**/**	0.6	0.3	1.1	15–24.9 g/day
William B.Kannel 1996*	**/**	**0.4**	**0.2**	**0.8**	**>25 g/day**
Yann Le Strat 2011*	15,884	**0.71**	**0.64**	**0.79**	**12 months moderate consumption**
Yann Le Strat 2011*	9,578	**0.6**	**0.49**	**0.66**	**12 months hazardous drinking**
Yann Le Strat 2011	1,484	0.8	0.59	1.1	12 months alcohol dependence
Yiti Liu 2024	37	1.34	0.5	3.56	<140 g/week
Yiti Liu 2024	104	3.87	1.65	9.06	140-279 g/week
Yiti Liu 2024	119	2.71	1.36	5.41	≥280 g/week

*Alcohol dosage/frequency is associated with arteriosclerosis. Bold font represents alcohol dosage/frequency is associated with artisosclerosis.

## 4 Discussion

This systematic review and meta-analysis assessed the effect of alcohol consumption on the risk of atherosclerosis. Data from 26 studies were analyzed to inform dietary guidelines, nutritional strategies, and preventive measures aimed at reducing atherosclerosis risk. The overall analysis did not show a significant direct effect of alcohol on atherosclerosis. However, subgroup analyses revealed key trends. Alcohol consumption was found to increase atherosclerosis risk in certain countries (Japan, South Korea, Brazil, and Denmark), study types (cohort and case–control studies), arterial locations (coronary arteries), diagnostic criteria (clinical diagnosis and computed tomography) and gender (female). Furthermore, the risk associated with alcohol intake varied with different levels of consumption. These findings suggest that alcohol’s impact on atherosclerosis may be influenced by environmental factors, study design, and individual characteristics. Therefore, considering alcohol consumption as a modifiable risk factor is crucial for the prevention and management of atherosclerosis.

The relationship between alcohol consumption and atherosclerosis may be shaped by differences in drinking habits, lifestyle, cultural factors, and alcohol intake across countries and regions. In countries such as Japan, South Korea, Brazil, and Denmark, alcohol consumption significantly increases the risk of atherosclerosis. However, no such association has been observed in countries like Nigeria, Britain, Spain, China, America, and Germany. This discrepancy may stem from variations in drinking cultures and habits. Our study indicates that different types of alcoholic beverages may not significantly differ in their impact on atherosclerosis risk. However, this conclusion is constrained by the limited number of relevant studies. Specifically, we included only 4 reports on beer, 3 on wine, and 2 on liquor. Therefore, the potential differential effects of various alcoholic beverages on atherosclerosis should not be overlooked. In Mediterranean countries like Spain and Italy, wine consumption is more common, and studies suggest that while beer and spirits are more strongly linked to all-cause mortality, the polyphenols in wine may offer protective cardiovascular benefits (47–49). Additionally, dietary patterns may differ between countries, such as in Nigeria and some Asian nations, where high-salt, high-sugar, and high-fat diets are prevalent. These factors could interact with alcohol consumption, further influencing cardiovascular health (50). To better understand this relationship, future research should focus on geographic and cultural differences and conduct multicenter studies across diverse regions.

Additionally, alcohol consumption appears to primarily increase the risk of coronary atherosclerosis, with a lesser effect on other arteries. Different biological mechanisms may regulate atherosclerosis in these regions, potentially contributing to biases in the outcomes. Firstly, coronary arteries, being medium-sized vessels, contain branching structures that are vital for blood supply to the heart. When blood flow is uneven or disturbed, arterial branches and collateral vessels are particularly prone to the development of atherosclerosis (51–53). Studies have shown that regions of coronary arteries with low wall shear stress (WSS) experience more significant plaque progression than areas with medium or high WSS. This promotes lipid deposition and the accumulation of inflammatory cells, advancing atherosclerosis and plaque development (54, 55). Compared to coronary arteries, other arteries may present distinct hemodynamic characteristics, which can influence lipid deposition and the accumulation of inflammatory cells, thereby affecting the formation of atherosclerosis. Secondly, under normal conditions, vascular endothelial cells play a crucial role in maintaining vascular homeostasis, regulating vascular tone, and balancing blood coagulation and fibrinolysis (56). However, alcohol consumption can disrupt this equilibrium, making endothelial cells more vulnerable to oxidative stress and inflammation, which in turn leads to endothelial dysfunction (7, 57). Once endothelial function is impaired, its protective effect on blood vessels is significantly reduced, creating conditions conducive to lipid deposition, prolonged inflammation, and the progression of atherosclerosis. The accumulation of lipids and extracellular matrix is particularly pronounced in the coronary arteries, thereby increasing the risk of heart attacks and coronary artery disease (58, 59). Finally, an individual’s genetic background, drinking habits, and other cardiovascular risk factors—such as hypertension and diabetes—significantly influence alcohol’s impact on various arteries. Genetic variations can cause individuals to metabolize and respond to alcohol differently, affecting the extent of vascular damage (60). Atherosclerosis of the coronary arteries is particularly closely related to the interaction of these factors, leading to more rapid progression of coronary artery disease and an increased risk of cardiovascular events. Overall, the increased risk of coronary artery atherosclerosis associated with alcohol consumption is closely linked to alterations in hemodynamics, lipid metabolism, and inflammatory responses. These factors interact to contribute to vascular damage and the progression of atherosclerosis. Understanding how these elements work together is essential for assessing the overall impact of alcohol on cardiovascular health.

Study also examined the relationship between alcohol consumption dose and frequency and the development of atherosclerosis. Overall, higher alcohol consumption is associated with an increased risk of atherosclerosis, but the effect varies depending on the amount consumed. Moderate alcohol intake (e.g., a small glass of red wine daily) may offer a protective benefit by raising high-density lipoprotein [HDL] cholesterol. However, exceeding a certain threshold may accelerate atherosclerosis. Previous studies have shown that moderate alcohol consumption is linked to a reduced risk of coronary artery disease [CAD], with the lowest risk observed at a daily intake of 36 grams (61). Ding found that alcohol consumption is related to cardiovascular events, but when patients consume about 105 grams of alcohol per week, both mortality and the risk of subsequent cardiovascular events are lower (62). Despite these findings, the “safe threshold” for alcohol intake remains unclear. Factors such as genetic background, lifestyle, and dietary habits can affect alcohol tolerance and may vary between individuals (63, 64). Therefore, future research should investigate the specific effects of moderate and excessive alcohol consumption on atherosclerosis and explore the underlying mechanisms.

We have found that women have a higher risk of developing atherosclerosis than men. Previous research has shown that the relationship between alcohol consumption and atherosclerosis differs significantly between genders, which is consistent with our conclusion. Pai et al. indirectly suggested that alcohol consumption may influence atherosclerosis risk through their investigation of the link between alcohol intake and inflammatory markers (65). In men, moderate alcohol consumption (1–2 drinks per day) is associated with lower levels of inflammatory markers, such as C-reactive protein and interleukin-6. In women, this association is more pronounced with half a drink per day. This suggests that moderate alcohol consumption may have different effects on atherosclerosis risk between men and women. Alcohol consumption on atherosclerosis risk factors may vary by gender and age. Wakabayashi’s research indicates that the impact of moderate alcohol consumption on atherosclerosis risk factors may vary by gender and age (66). In men, moderate alcohol consumption may reduce total cholesterol levels and increase HDL-C levels, thereby positively affecting atherosclerosis risk factors. In women, these beneficial effects are more evident in younger individuals but may diminish with age. Georgescu found that moderate alcohol consumption may offer some protection to cardiovascular health, although this protective effect is more pronounced in men (54). In men, alcohol consumption may exert positive effects by reducing total cholesterol and increasing HDL-C levels. However, in women, these benefits tend to diminish with advancing age.

Finally, the observed heterogeneity may also stem from differences in study design and diagnostic criteria. Cohort and case–control studies have shown a significant association between alcohol consumption and atherosclerosis, offering advantages over cross-sectional studies. Cohort studies prospectively track participants to observe the long-term relationship between alcohol consumption and atherosclerosis development, better assessing causality (67–69). In contrast, case–control studies compare individuals with and without atherosclerosis, examining their alcohol consumption history to control for confounding factors and enhance result accuracy (70, 71). Cross-sectional studies provide merely a snapshot of the situation at a particular moment, making it difficult to establish causality and increasing their susceptibility to confounding (72). Moreover, different diagnostic criteria, such as clinical diagnosis, computed tomography, and ultrasound, may lead to differences in atherosclerosis detection and classification, affecting the observed associations. Therefore, to accurately interpret study findings, it is essential to consider these factors when evaluating the relationship between alcohol consumption and atherosclerosis.

Despite offering valuable insights into the relationship between alcohol consumption and atherosclerosis, our study has several limitations. First, significant variability across studies in factors such as diet, types of alcoholic beverages, alcohol content, and lifestyle habits may contribute to inconsistent results, affecting the reliability of conclusions. Second, the biological mechanisms underlying atherosclerosis may differ based on its location in the body, a factor that cannot be ruled out and could introduce bias. Third, publication bias is a common concern, as studies with negative or non-significant results are less likely to be published, which may lead to an overestimation of alcohol’s impact on atherosclerosis. Fourth, meta-analyses rely on aggregated data, lacking detailed individual-level analysis, which hinders a thorough understanding of how factors like alcohol consumption frequency and duration relate to disease risk (73). Furthermore, many of the studies included in our analysis are observational, with issues such as small sample sizes and uneven data distribution, which could compromise the robustness of the results. Finally, the time-lag effect may prevent timely reflection of the latest research developments.

The subgroup analysis by gender in this study was conducted based on aggregated data, and this approach may not accurately estimate the expected interactions. According to the research by Fisher et al. (74), conducting subgroup analysis using only aggregated data may lead to ecological bias, thus affecting the reliability of the results. An interaction analysis is needed to evaluate the robustness of the article. However, due to the diverse types of studies involved in our article, such as cross - sectional studies, cohort studies, and case - control studies, and the fact that some of the included articles were published relatively long ago, it was not possible to obtain precise control and treatment group samples. With only the odds ratio (OR) values and 95% confidence intervals for alcohol consumption, there are certain limitations. Although we have tried to control potential confounding factors through statistical methods, this bias may not be completely eliminated. We have also made multiple attempts, but ultimately, we are still unable to complete this interaction analysis using the available aggregate data. Therefore, the results of the gender subgroup analysis should be regarded as exploratory and mainly used for hypothesis - generation rather than as a final conclusion. Future studies should consider using individual participant data for more accurate interaction analysis to verify these preliminary findings.

In conclusion, when interpreting meta-analytic findings on alcohol consumption and atherosclerosis, it is crucial to consider heterogeneity, publication bias, and data limitations to avoid drawing misleading or overly simplistic conclusions. These factors emphasize the need for more rigorous, well-designed studies to confirm our findings.

## 5 Conclusion

These findings suggest that the impact of alcohol on atherosclerosis may be influenced by environmental factors, study designs, and individual characteristics. Future research should focus on investigating the mechanisms by which alcohol consumption—considering factors like amount, frequency, and type—affects atherosclerosis. In particular, more studies are needed to explore alcohol consumption thresholds and the underlying biological mechanisms. Given that the effects of alcohol on atherosclerosis may depend on a range of factors, such as genetic background, diet, and lifestyle, a more individualized approach is necessary. By delving deeper into these variables, we aim to offer more precise clinical guidance to reduce the risk of atherosclerosis associated with alcohol consumption.

## Data Availability

The original contributions presented in the study are included in the article/Supplementary material, further inquiries can be directed to the corresponding author.
