# Supplementary material for: Impact of alcohol consumption on atherosclerosis: a systematic review and meta-analysis
Source: Front Nutr. 2025 Apr 30;12:1563759. doi: 10.3389/fnut.2025.1563759 (PMC12075366; doi:10.3389/fnut.2025.1563759)
Supplement: Supplementary file 1 [file Table_1.docx]

**Impact of Alcohol Consumption on atherosclerosis: A Systematic Review and Meta-analysis**

**Supplemental Table 1.** Search strategies in Pubmed（3837）

| Search |  | Query |
| --- | --- | --- |
| #1  #2  #3 |  | ((((((Alcohol Drinking[MeSH Major Topic]) OR (Ethanol[MeSH Major Topic])) OR (Alcohol Consumption[MeSH Major Topic])) OR (Alcohol Intake[MeSH Major Topic])) OR (Beer[MeSH Major Topic])) OR (Wine[MeSH Major Topic])) OR (Liquor[MeSH Major Topic]) OR Alcohol Drinking OR Ethanol OR Alcohol Consumption OR Alcohol Intake OR Beer OR Wine OR Liquor  (((((((Atheroscleroses[MeSH Major Topic]) OR (Atherogenesis[MeSH Major Topic])) OR (Atherogeneses[MeSH Major Topic])) OR (Limb atherosclerosis[MeSH Major Topic])) OR (Coronary atherosclerosis[MeSH Major Topic])) OR (Carotid atherosclerosis[MeSH Major Topic])) OR (Intracranial Arterioscleroses[MeSH Major Topic])) OR (Cerebral Arteriosclerosis[MeSH Major Topic]) OR Atheroscleroses OR Atherogenesis OR Atherogeneses OR Limb atherosclerosis OR Coronary atherosclerosis OR Carotid atherosclerosis OR Intracranial Arterioscleroses OR Cerebral Arteriosclerosis  #1 AND #2 |

**Supplemental Table 2.** Search strategies in Embase（376）

| Search |  | Query |
| --- | --- | --- |
| #1  #2  #3 |  | 'alcohol drinking':ti,ab,kw OR ethanol:ti,ab,kw OR 'alcohol consumption':ti,ab,kw OR 'alcohol intake':ti,ab,kw OR beer:ti,ab,kw OR wine:ti,ab,kw OR liquor:ti,ab,kw  atheroscleroses:ti,ab,kw OR atherogenesis:ti,ab,kw OR atherogeneses:ti,ab,kw OR 'limb atherosclerosis':ti,ab,kw OR 'coronary atherosclerosis':ti,ab,kw OR 'carotid atherosclerosis':ti,ab,kw OR 'intracranial arterioscleroses':ti,ab,kw OR 'cerebral arteriosclerosis':ti,ab,kw  #1 AND #2 |

**Supplemental Table 3.** Search strategies in Cochrane（99）

| Search |  | Query |
| --- | --- | --- |
| #1  #2  #3 |  | (Alcohol Drinking) OR (Ethanol) OR (Alcohol Consumption) OR (Alcohol Intake) OR (Beer) OR (Wine) OR (Liquor)  (Atheroscleroses) OR (Atherogenesis) OR (Atherogeneses) OR (Limb atherosclerosis) OR (Coronary atherosclerosis) OR (Carotid atherosclerosis) OR (Intracranial Arterioscleroses) OR (Cerebral Arteriosclerosis)  #1 AND #2 |

**Supplemental Table 4.** Search strategies in Web of Science（375）

| Search |  | Query |
| --- | --- | --- |
| #1  #2  #3 |  | ((((((TI=(Alcohol Drinking)) OR TI=(Ethanol)) OR TI=(Alcohol Consumption)) OR TI=(Alcohol Intake)) OR TI=(Beer)) OR TI=(Wine)) OR TI=(Liquor)  (((((((TS=(Atheroscleroses)) OR TS=(Atherogenesis)) OR TS=(Atherogeneses)) OR TS=(Limb atherosclerosis)) OR TS=(Coronary atherosclerosis)) OR TS=(Carotid atherosclerosis)) OR TS=(Intracranial Arterioscleroses)) OR TS=(Cerebral Arteriosclerosis)  #1 AND #2 |

**Supplemental Table 5.** AHRQ Evaluation

| Inclusion in the study | 1 | 2 | 3 | 4 | 5 | 6 | 7 | 8 | 9 | 10 | 11 |
| --- | --- | --- | --- | --- | --- | --- | --- | --- | --- | --- | --- |
| Adeleye Dorcas Omisore 2018 | yes | yes | unclear | unclear | no | yes | yes | unclear | no | no | no |
| Akihiko Krtamur 1998 | yes | unclear | unclear | unclear | no | yes | yes | unclear | yes | yes | yes |
| Annie Britton 2004 | yes | yes | unclear | unclear | no | yes | yes | yes | no | no | no |
| Belén Moreno-Franco 2020 | yes | yes | unclear | unclear | no | yes | yes | unclear | yes | yes | no |
| Chun Zhang 2022 | yes | yes | unclear | unclear | no | yes | yes | unclear | yes | yes | no |
| Dong Hyun Sinn 2014 | yes | yes | unclear | yes | no | yes | yes | unclear | yes | yes | no |
| Dwayne Reed 1991 | yes | unclear | unclear | unclear | no | yes | yes | unclear | yes | yes | no |
| Flávio D.Fuchs 2004 | yes | unclear | unclear | unclear | no | yes | yes | unclear | yes | yes | yes |
| Franziska K Bishop 2009 | yes | unclear | unclear | unclear | no | yes | yes | unclear | yes | yes | no |
| Hermann Brenner 2001 | yes | yes | unclear | unclear | no | yes | yes | yes | yes | yes | no |
| Janne Tolstrup 2006 | yes | unclear | unclear | unclear | no | yes | yes | yes | yes | yes | yes |
| Jeanne K.Tofferi 2004 | yes | yes | unclear | unclear | no | yes | no | yes | no | yes | no |
| Jurgen T.Rehm 1997 | yes | unclear | unclear | unclear | no | yes | no | yes | no | yes | no |
| Koichi Handa 1990 | yes | yes | unclear | unclear | no | yes | no | yes | no | yes | no |
| Marcello Ricardo Paulista Markus 2015 | yes | unclear | unclear | unclear | no | yes | yes | yes | yes | yes | no |
| Mark J.Pletcher 2005 | yes | yes | unclear | unclear | no | yes | no | yes | no | yes | no |
| Michiko Fujisawa 2008 | yes | yes | unclear | unclear | no | yes | yes | unclear | yes | yes | no |
| Mihaela Tanasescu 2001 | yes | unclear | unclear | unclear | no | yes | no | yes | no | no | yes |
| Qi Cheng 2022 | yes | yes | unclear | unclear | no | yes | yes | yes | yes | yes | yes |
| Tianyu Zhou 2023 | yes | yes | unclear | unclear | no | yes | no | no | no | no | no |
| Umed A.Ajani 2015 | yes | unclear | unclear | unclear | no | yes | no | no | no | no | yes |
| William B.Kannel 1996 | yes | unclear | unclear | unclear | no | no | no | unclear | no | no | no |
| Xiaohuan Chen 2024 | yes | yes | unclear | unclear | no | yes | yes | yes | yes | yes | no |
| Yann Le Strat 2011 | yes | unclear | unclear | unclear | no | no | no | no | no | no | no |
| Yinze Ji 2024 | yes | yes | unclear | unclear | no | yes | no | yes | no | no | no |
| Yiti Liu 2024 | yes | yes | unclear | unclear | no | yes | yes | yes | yes | yes | no |

Note: 1. Whether the source of the data is clearly defined; 2. Whether the inclusion and exclusion criteria for the exposed and non-exposed groups are listed or referenced from previous publications; 3. Whether the time phase for identifying patients is given; 4. Whether the study subjects are continuous if not from a population source; 5. Whether the evaluator's subjective factors mask other aspects of the study subjects; 6. Whether any assessments for ensuring quality are described; 7. Whether the reasons for excluding any patients from the analysis are explained; 8. Whether measures for evaluating and/or controlling confounding factors are described; 9. If possible, whether the handling of missing data in the analysis is explained; 10. Whether the response rate of patients and the completeness of data collection are summarized; 11. If there is a follow-up, whether the percentage of expected incomplete data from patients or the follow-up is identified


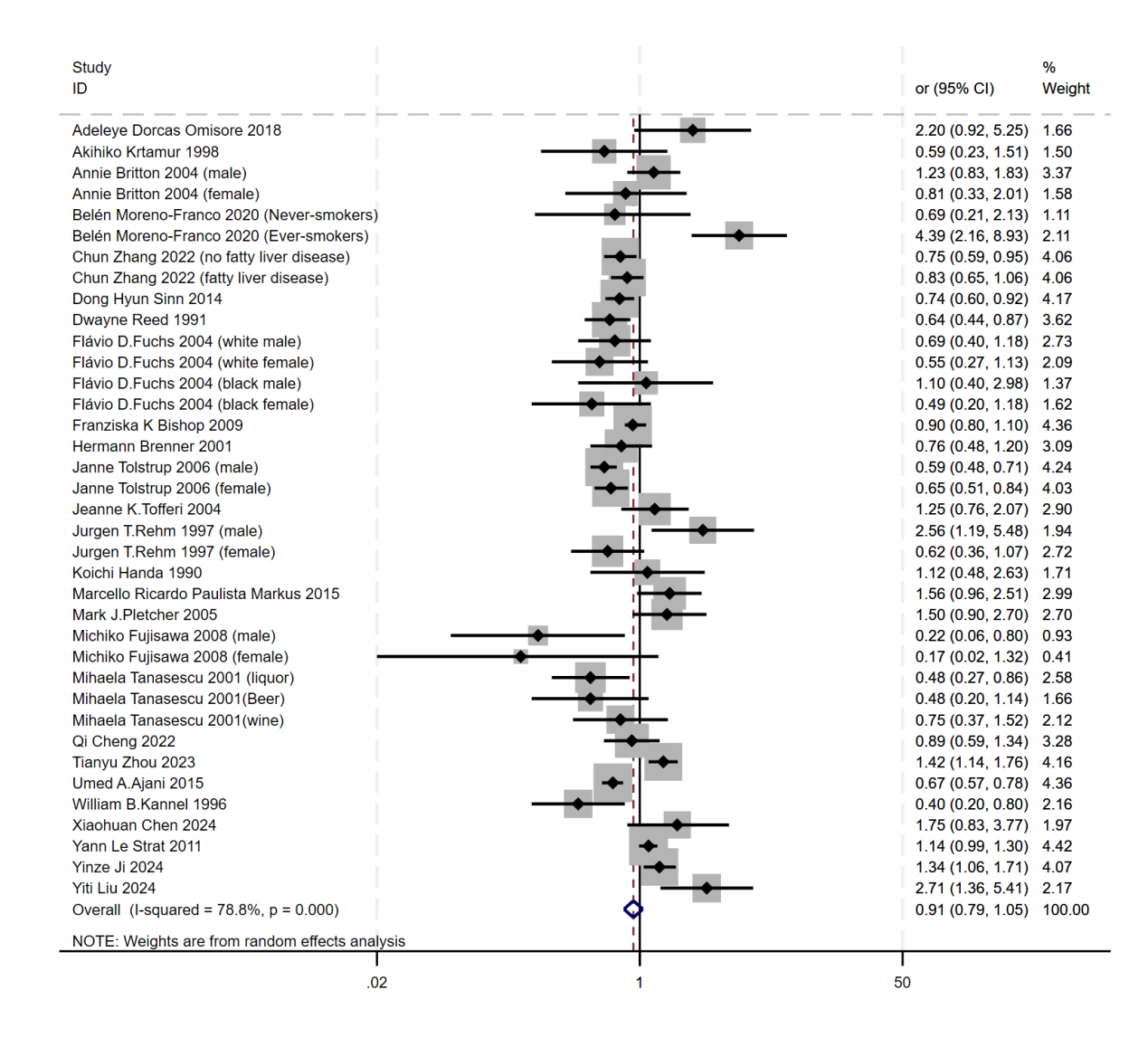


**Supplemental Figure 1.** Forest diagram of the relationship between alcohol consumption and arteriosclerosis


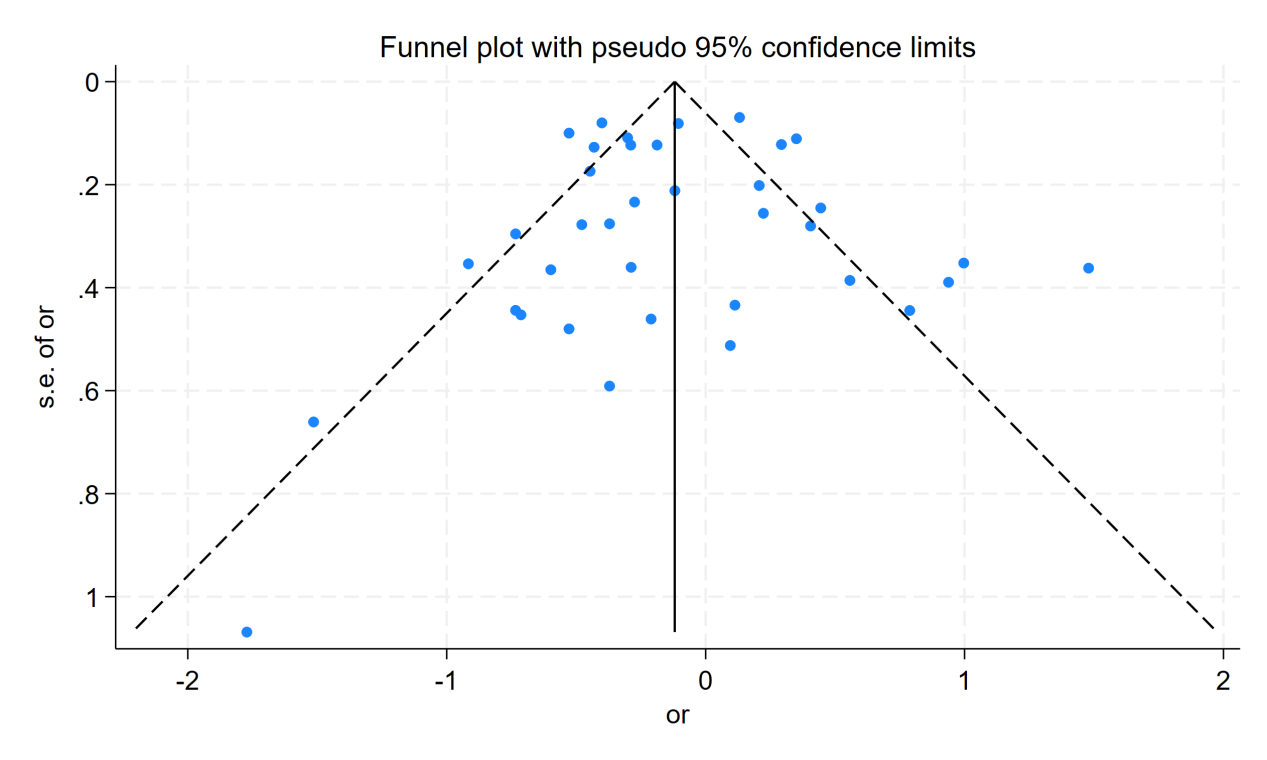


**Supplemental Figure 2**. Funnel Plot of the Relationship Between Alcohol Consumption and Atherosclerosis.


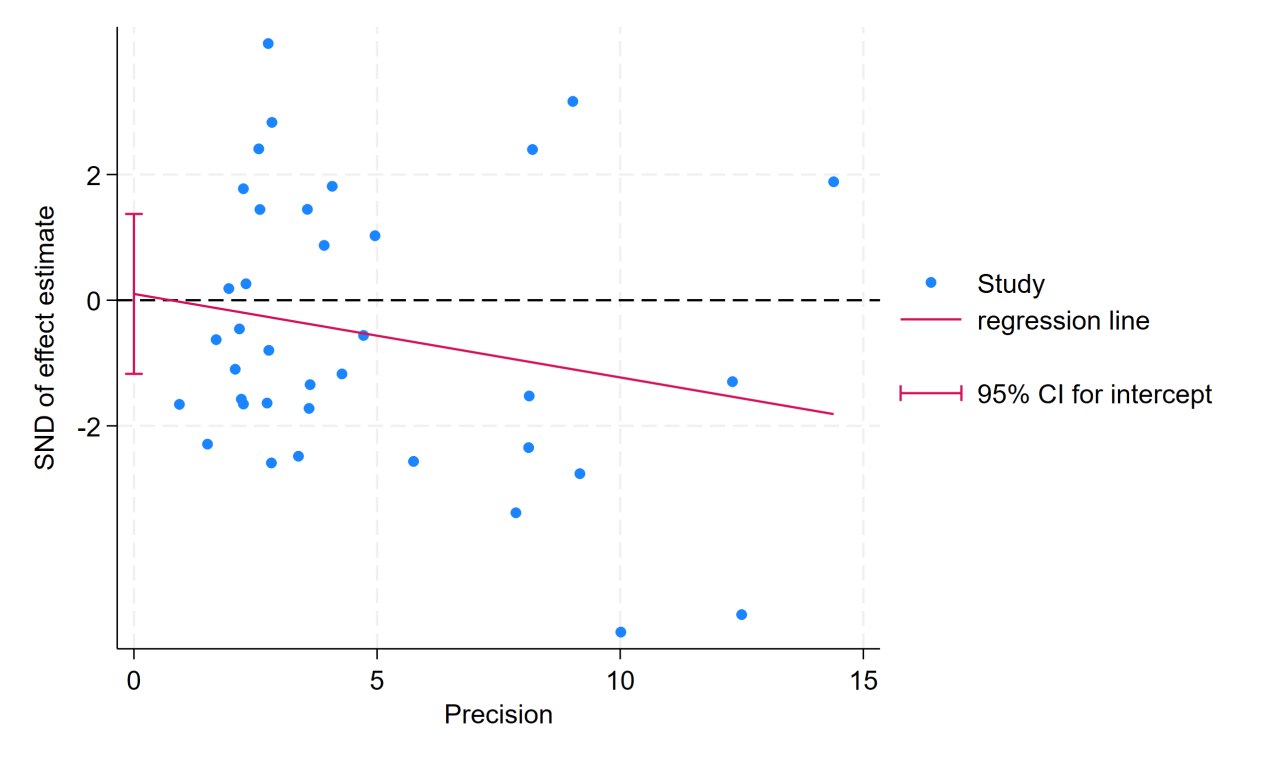


**Supplemental Figure 3**. Egger's Test for the Relationship Between Alcohol Consumption and Atherosclerosis.

**
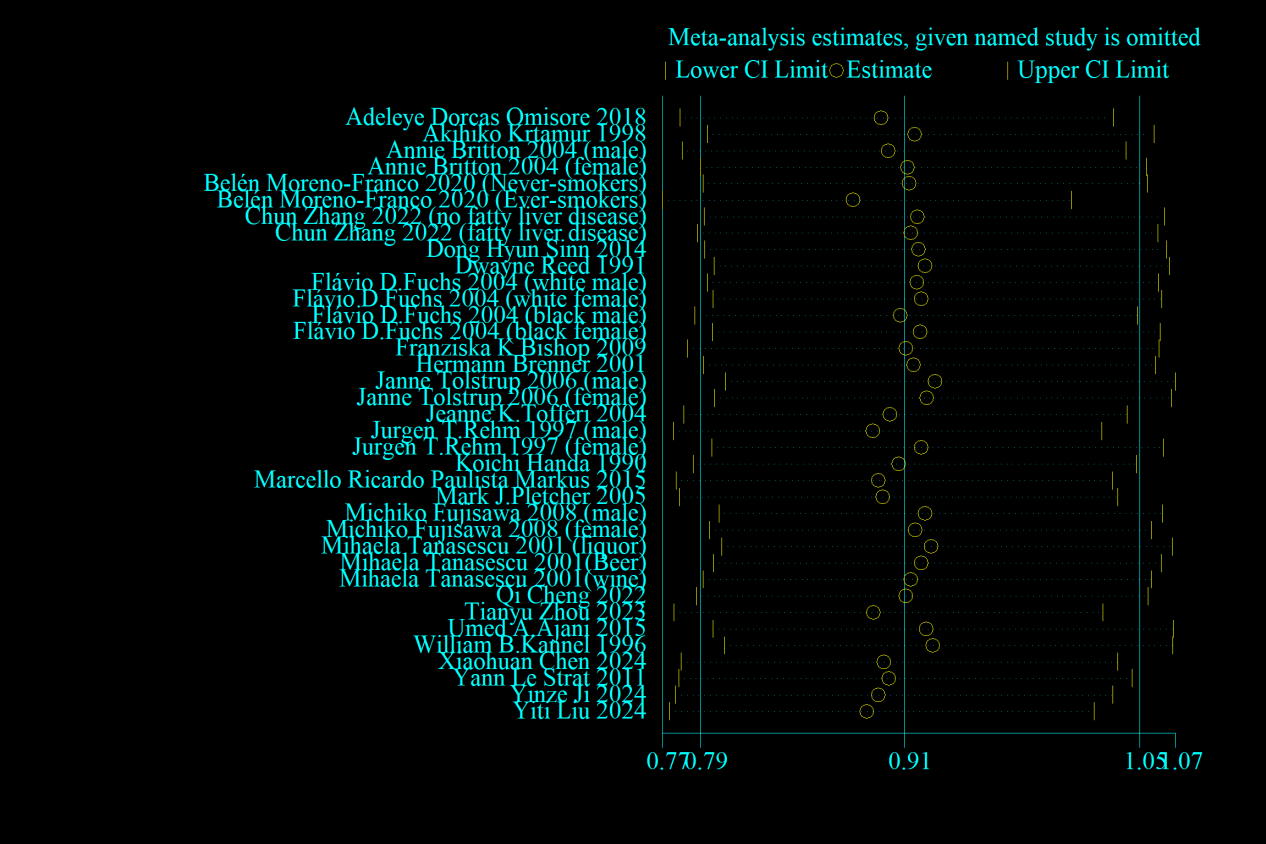
**

**Supplemental Figure 4**.Sensitivity analysis of the relationship between alcohol consumption and arteriosclerosis

**
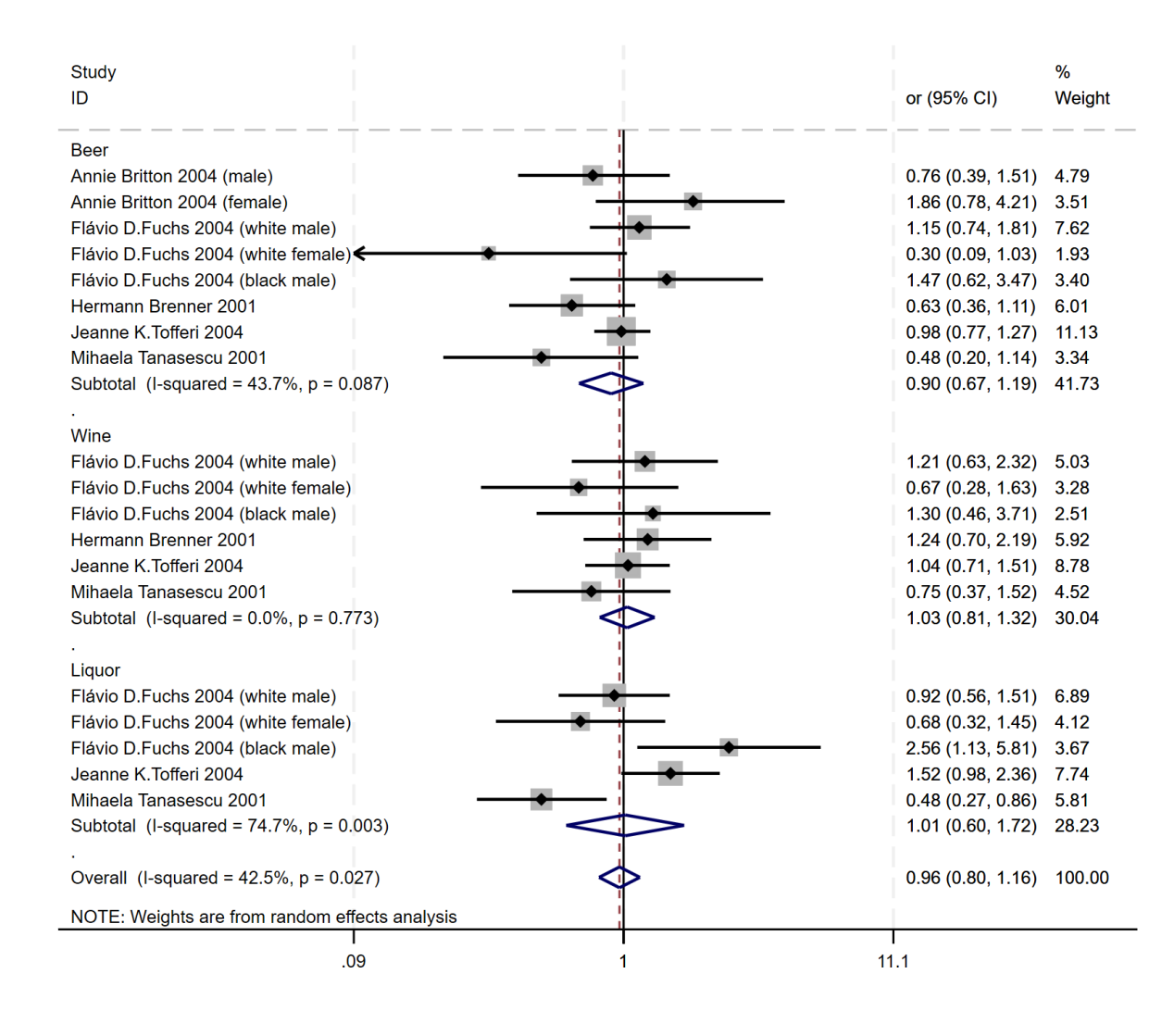
**

**Supplemental Figure 5.** Subgroup analysis of the relationship between alcohol consumption and arteriosclerosis - forest plot of alcohol types
