# Supplementary material for: Impact of alcohol consumption on atherosclerosis: a systematic review and meta-analysis
Source: Front Nutr. 2025 Apr 30;12:1563759. doi: 10.3389/fnut.2025.1563759 (PMC12075366; doi:10.3389/fnut.2025.1563759)
Supplement: Supplementary file 2 [file Table_2.docx]

| **Section and Topic** | **Item #** | **Checklist item** | **Location where item is reported** |
| --- | --- | --- | --- |
| **TITLE** | | |  |
| Title | 1 | Impact of Alcohol Consumption on atherosclerosis: A Systematic Review and Meta-analysis |  |
| **ABSTRACT** | | |  |
| Abstract | 2 | Introduction: Atherosclerosis, a chronic vascular disease, impacts various arterial systems, such as the coronary, carotid, cerebral, renal, and peripheral arteries. Dietary factors, especially alcohol consumption, significantly contribute to the progression of atherosclerosis. However, systematic evaluations of alcohol's impact on atherosclerosis are still limited. This study investigates the impact of alcohol consumption on atherosclerosis via meta-analysis and assesses the moderating effects of drinking frequency, gender, and other factors. Methods: By December 2024, a comprehensive literature search was conducted across PubMed, Embase, Cochrane, and Web of Science databases. Studies evaluating the relationship between alcohol consumption and atherosclerosis were rigorously selected and assessed for quality. The study protocol was registered with the INPLASY database. Data extraction and statistical analysis were conducted using STATA 18.0 software. A total of 26 studies involving 326,513 patients across 10 countries were included. Considering that different biological mechanisms may regulate atherosclerosis in different arterial locations, we conducted subgroup analyses to explore differences in country, study type, arterial site, diagnostic criteria, type of alcohol, and gender. Result: The results show that the overall analysis did not show a significant promoting effect of alcohol consumption on the development of atherosclerosis (OR=0.92, 95% CI 0.80-1.06, P =0.227). Subgroup analyses revealed several important trends. Alcohol consumption may increase the risk of atherosclerosis in specific countries (Japan, South Korea, Brazil, and Denmark), types of studies (cohort and case-control studies), arterial locations (coronary arteries), and diagnostic criteria (clinical diagnosis and computed tomography). Interestingly, we found that alcohol consumption may increase the risk of atherosclerosis in women. Furthermore, varying levels of alcohol consumption appear to result in differing risks of the disease. Conclusions: The impact of alcohol consumption on atherosclerosis is not singular and may interact with multiple factors, including environmental factors, lesion location, and individual characteristics. | Abstract |
| **INTRODUCTION** | | |  |
| Rationale | 3 | Atherosclerosis is a chronic vascular condition characterized by lipid deposition in the arterial intima, smooth muscle cell proliferation, and increased fibrous tissue, leading to plaque formation, arterial narrowing, hardening, and blood flow obstruction. Dietary factors, particularly alcohol consumption, play a significant role in the onset and progression of atherosclerosis. | 1Introduction |
| Objectives | 4 | This study investigates the relationship between alcohol consumption and the risk of atherosclerosis through a systematic review and meta-analysis. It specifically examines the type of alcoholic beverage, the various sites of atherosclerosis (including the coronary, carotid, and peripheral arteries), and regional differences across countries. The results aim to provide evidence-based guidance for the clinical management of atherosclerosis, supporting the development of precision medicine and personalized treatment strategies. | 1Introduction |
| **METHODS** | | |  |
| Eligibility criteria | 5 | To evaluate the relationship between alcohol consumption and atherosclerosis, only studies involving patients diagnosed with atherosclerosis were included. There were no restrictions on study type; however, studies were required to report multivariable-adjusted statistical measures, such as relative risk (RR), odds ratio (OR), hazard ratio (HR), and 95% confidence intervals (CI). To maintain scientific rigor, only cohort, case-control, and cross-sectional studies were deemed eligible for inclusion in the analysis. | 2.3  Inclusion Criteria |
| Information sources | 6 | We conducted a search across four major databases: PubMed, Embase, Cochrane, and Web of Science to ensure comprehensive coverage of relevant literature. The search focused on studies examining the relationship between alcohol consumption and atherosclerosis, spanning from the inception of each database through December 2024.  To ensure methodological transparency and rigor, this meta-analysis followed the Meta-analysis of Observational Studies in Epidemiology (MOOSE) guidelines and the Preferred Reporting Items for Systematic Reviews and Meta-Analyses (PRISMA) statement. These guidelines provide a standardized framework for conducting and reporting systematic reviews and meta-analyses. Additionally, the study protocol has been registered with the International Platform of Registered Systematic Review and Meta-analysis Protocols (INPLASY) database (registration number: INPLASY202510031), further enhancing transparency and minimizing the risk of bias. | 2.2  Literature search strategy  2.6 Literature Screening and Data Extraction |
| Search strategy | 7 | The search terms included: "Alcohol Drinking," "Ethanol," "Alcohol Consumption," "Alcohol Intake," "Beer," "Wine," "Liquor," "Atherosclerosis," "Atherogenesis," "Atherogeneses," "Limb Atherosclerosis," "Coronary Atherosclerosis," "Carotid Atherosclerosis," "Intracranial Arteriosclerosis," and "Cerebral Arteriosclerosis." This systematic search strategy was designed to capture a broad range of relevant studies, thereby enhancing the reliability and comprehensiveness of the findings. | 2.2  Literature search strategy |
| Selection process | 8 | Inclusion Criteria: To assess the relationship between alcohol consumption and atherosclerosis, the analysis included only studies that involved patients diagnosed with atherosclerosis. There were no restrictions based on the type of study; however, it was mandatory for the studies to report multivariable-adjusted statistical measures, including relative risk (RR), odds ratio (OR), hazard ratio (HR), and 95% confidence intervals (CI). To uphold scientific rigor, only cohort, case-control, and cross-sectional studies were considered eligible for inclusion in the analysis.  Exclusion Criteria: The following types of studies were excluded from the analysis: studies not involving patients diagnosed with atherosclerosis; studies whose results did not align with the inclusion criteria; reviews, case reports, survey analyses, conference abstracts, and irrelevant literature; and duplicate publications.  Two reviewers worked independently on the screening process, managing the literature with the help of Endnote software. | 2.3 Inclusion Criteria  2.4 Exclusion Criteria |
| Data collection process | 9 | Based on predefined criteria, two reviewers (Song and Hu) independently screened the titles, abstracts, and full texts of articles retrieved from various databases to assess their eligibility for inclusion. In case of disagreement, the original articles were re-examined, and consensus was reached through discussion. | 2.5 Literature Screening and Data Extraction |
| Data items | 10a | Relevant data were extracted from the selected studies, including author names, publication year, country, study type, alcohol type, atherosclerosis site, diagnostic criteria, sample size, age, gender. | 2.5 Literature Screening and Data Extraction |
|  | 10b | Relevant data were extracted from the selected studies, including odds ratio, lower confidence interval, upper confidence interval, grouping basis and covariate adjustments | 2.5 Literature Screening and Data Extraction |
| Study risk of bias assessment | 11 | We conducted a meta-analysis using STATA 18.0 software. To assess publication bias, we generated funnel plots and conducted Egger's test, examining potential biases in studies on alcohol consumption and atherosclerosis | 2.7 Statistical analysis |
| Effect measures | 12 | The effect measure used in our meta-analysis for the impact of alcohol consumption is the (odds ratio, lower confidence interval, upper confidence interval). | 2.5 Literature Screening and Data Extraction |
| Synthesis methods | 13a | To determine which studies were eligible for each synthesis, we followed a systematic approach based on predefined inclusion and exclusion criteria. Initially, we screened studies to ensure they involved patients diagnosed with atherosclerosis. We then assessed whether the studies were cohort, case-control, or cross-sectional designs. Additionally, we checked if the studies reported multivariable-adjusted statistical measures such as relative risk (RR), odds ratio (OR), and 95% confidence intervals (CI). Studies meeting these criteria were further evaluated for subgroup analyses planned, such as country, study type, arterial site, diagnostic criteria, type of alcohol, and gender. This rigorous process ensured the scientific relevance and robustness of the included studies. | 2.3 Inclusion Criteria  2.4 Exclusion Criteria  2.5 Literature Screening and Data Extraction  3.2  Results of Included Study Characteristics |
|  | 13b | To prepare the data for presentation and synthesis, we employed several methods. For missing summary statistics, we attempted to contact the original study authors to obtain complete information. When this was not possible, we made reasonable estimates based on available data and statistical methods. We standardized the format of effect size indicators (e.g., OR and CI) across studies to ensure consistency for pooled analysis. Data were also normalized to account for potential differences in units or measurement methods, enhancing comparability and accuracy in our analyses. | 2.7 Statistical analysis |
|  | 13c | We used a combination of tabular and graphical methods to present the results of individual studies and syntheses clearly. We compiled key study characteristics, including authors, publication years, countries, study types, and effect size indicators (OR and 95% CI), into detailed tables for easy comparison and reference. Forest plots were utilized to visually represent the effect sizes and confidence intervals of each study, while funnel plots assessed publication bias. Additionally, we created subgroup-specific charts to illustrate differences in effect sizes across various categories, providing intuitive visual summaries of the data. | 2.7 Statistical analysis  3.2  Results of Included Study Characteristics |
|  | 13d | For synthesizing results, we primarily used the random-effects model (RE) due to significant heterogeneity among studies (I² = 78.8%), indicating that a fixed-effects model was inappropriate. We conducted the meta-analysis using STATA 18.0 software, calculating effect size indicators (OR) and their 95% confidence intervals to assess the relationship between alcohol consumption and atherosclerosis. Heterogeneity was quantified using the I² statistic, and publication bias was assessed using Egger's test. Sensitivity analyses were also performed to validate the robustness of our findings. | 2.7 Statistical analysis  3.3.1 Relationship between alcohol consumption and atherosclerosis |
|  | 13e | To explore potential causes of heterogeneity among study results, we conducted subgroup analyses based on factors such as country, study type, arterial site, diagnostic criteria, type of alcohol, and gender. By dividing studies into these subgroups, we identified significant differences in the relationship between alcohol consumption and atherosclerosis across various categories. For instance, notable variations were observed in specific countries (e.g., Japan, South Korea, Brazil, and Denmark) and study types (cohort and case-control studies). These analyses helped elucidate sources of heterogeneity and guided future research directions. | 3.2  Results of Included Study Characteristics |
|  | 13f | To assess the robustness of our synthesized results, we performed sensitivity analyses by sequentially excluding individual studies and observing changes in the overall effect size. The results remained consistent, indicating high robustness of our findings. Additionally, we tested different data handling methods and model selections, further confirming the reliability of our results. | 2.7 Statistical analysis |
| Reporting bias assessment | 14 | We assessed the risk of bias due to missing results using Egger's test and funnel plots. The funnel plot displays the relationship between the effect size (OR) of each study and its standard error (SE). The asymmetric distribution of the points, especially some on the right side located outside the pseudo 95% confidence intervals of the funnel plot, may indicate the presence of publication bias. Egger's Test (P = 0.874, > 0.05) shows the relationship between the precision of each study and the standardized normal deviation (SND) of the effect estimate. The regression line indicates a negative correlation between the effect estimate and precision, which may suggest the presence of a small-study effect. The 95% confidence interval (CI) in the plot is used to assess the uncertainty of the intercept | 2.7 Statistical analysis  3.3.1 Relationship between alcohol consumption and atherosclerosis |
| Certainty assessment | 15 | To assess the certainty of the evidence, we considered factors such as study design, sample size, quality, and potential confounding factors. We evaluated study quality using criteria recommended by the Agency for Healthcare Research and Quality (AHRQ), focusing on aspects like clear data sources, well-defined inclusion and exclusion criteria, and control of confounding factors. While most studies performed well in these areas, the presence of heterogeneity and potential publication bias led us to approach our conclusions with caution. Additionally, the observational nature of most studies limited our confidence in inferring causality. | 2.7 Statistical analysis  3.2Results of Included Study Characteristics |
| **RESULTS** | | |  |
| Study selection | 16a | 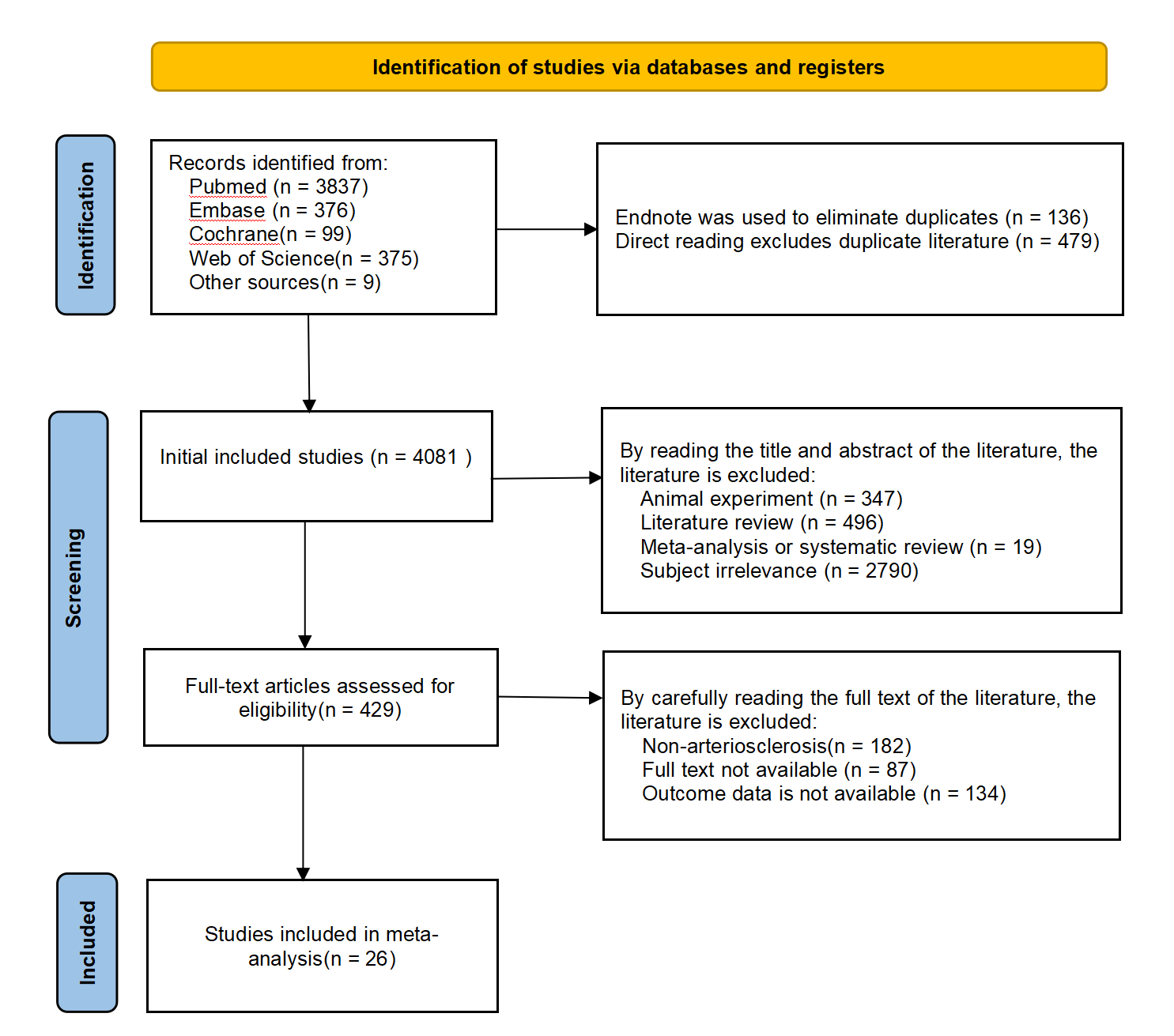 | 3.1 Results of Literature Search |
|  | 16b | Identification:  To manage the volume of records, we utilized Endnote software to eliminate duplicates, which reduced the number to 136 studies. Additionally, direct reading helped us exclude an additional 479 duplicate literatures.  Screening:  This screening process led to the exclusion of several types of literature: 347 animal experiments, 496 literature reviews, 19 meta-analyses or systematic reviews, and 2790 studies deemed irrelevant to the subject matter.  Eligibility Assessment:  Full-text articles were carefully reviewed, leading to further exclusions based on specific criteria: 182 studies were excluded because they did not focus on atherosclerosis, 87 studies were excluded due to the unavailability of the full text, and 134 studies were excluded because they did not provide the necessary outcome data. | 3.1Results of Literature Search |
| Study characteristics | 17 | In this study, a total of 26 studies were included, encompassing 326,513 patients from 10 different countries. These studies comprised 2 case-control studies, 10 cohort studies, and 14 cross-sectional studies. The types of alcohol consumption covered in these studies primarily included beer, spirits, and wine. The locations affected by atherosclerosis varied, including the femoral artery, aorta, coronary artery, and carotid artery. The age range of the patients was broad, with all participants being over 18 years old. Among the 25 studies, there were 215,986 male patients and 110,527 female patients.  Citing each included study and presenting its characteristics:  1. Adeleye Dorcas Omisore (2018, Nigeria): This cross-sectional study involved 162 patients, focusing on carotid artery examination primarily through ultrasound.  2. Akihiko Krtamur (1998, Japan): This cross-sectional study included 8,476 patients, investigating the clinical diagnosis of coronary artery disease.  3. Annie Britton (2004, UK): This cohort study comprised 10,308 participants, examining the impact of beer consumption on the coronary artery.  4. Belén Moreno-Franco (2020, Spain): This cross-sectional study involved 2,099 male participants who never smoked, focusing on the ultrasound examination results of the femoral artery.  5. Chun Zhang (2022, China): This cross-sectional study included 47,063 patients, examining the ultrasound examination results of the carotid artery.  6. Dong Hyun Sinn (2014, South Korea): This cross-sectional study included 2,280 patients, examining the carotid artery through ultrasound.  7. Dwayne Reed (1991, Japan): This cohort study involved 7,591 patients, studying the clinical diagnosis of the coronary artery.  8. Flávio D.Fuchs (2004, Brazil): This cohort study included 14,506 patients, investigating the effects of different types of alcoholic beverages on the coronary artery.  9. Franziska K Bishop (2009, USA): This cross-sectional study included 1,306 patients, focusing on the clinical diagnosis of the coronary artery.  10. Hermann Brenner (2001, Germany): This case-control study included 791 patients, examining the coronary artery through coronary angiography.  Please note that these are examples of some of the studies included. Each study has its unique characteristics, including study design, participant characteristics, intervention measures, and outcome measurement methods, all of which have been described and discussed in detail in our systematic review and meta-analysis. | 3.2  Results of Included Study Characteristics |
| Risk of bias in studies | 18 | In this study, we assessed the quality of the 26 included studies using the criteria recommended by the Agency for Healthcare Research and Quality (AHRQ). The assessment covered several aspects, including whether the data source was clearly defined, whether the inclusion and exclusion criteria were clear, whether the study subjects were selected consecutively, whether there was potential for assessor bias, whether quality assurance measures were described, whether reasons for excluding patients were explained, and whether confounding factors were controlled. Each of the 11 items for each study was evaluated as "yes," "no," or "unclear."  Among the included studies, the majority performed well in terms of data source clarity, inclusion and exclusion criteria, and control of confounding factors, providing a certain level of quality assurance for the study results. However, some studies had shortcomings in describing quality assurance measures and handling missing data, which may introduce potential bias. For example, some studies did not provide detailed explanations on how to avoid the impact of assessor bias on the study results, which could affect the objectivity of the findings. Moreover, studies with small sample sizes or uneven data distribution may not have been able to fully control all potential confounding factors, thus affecting the accuracy of the results. Nevertheless, through comprehensive analysis and sensitivity analysis, we believe that the impact of these biases on the overall study results is limited, and the study results still have a certain level of reliability and reference value. | 3.2  Results of Included Study Characteristics  3.3.1 Relationship between alcohol consumption and atherosclerosis |
| Results of individual studies | 19 | In this study, we have meticulously presented the results of the 26 individual studies included in our analysis. For each study, we provided detailed information, including the study's basic characteristics (such as author, publication year, country, study type, type of alcohol consumption, site of atherosclerosis, diagnostic criteria, sample size, age, and gender), as well as the effect estimates and their precision (e.g., odds ratio [OR], 95% confidence interval [CI]) regarding the relationship between alcohol consumption and atherosclerosis.  For example, in the study by Adeleye Dorcas Omisore (2018, Nigeria), a cross-sectional study involving 162 patients primarily examined carotid atherosclerosis through ultrasound. The results showed no significant association between alcohol consumption and the risk of carotid atherosclerosis . Similarly, Akihiko Krtamur (1998, Japan) conducted a cross-sectional study with 8,476 patients focusing on the clinical diagnosis of coronary artery disease. The findings indicated no significant relationship between alcohol consumption and coronary artery atherosclerosis. In the cohort study by Annie Britton (2004, UK), which included 10,308 participants examining the impact of beer consumption on the coronary artery, no significant association was found.  These results highlight the variability in effect estimates across different studies regarding the relationship between alcohol consumption and atherosclerosis. While some studies suggest that alcohol consumption may increase the risk of atherosclerosis, others do not show a significant association. This discrepancy may be attributed to various factors, including study design, sample characteristics, type and amount of alcohol consumption, and the site of atherosclerosis. By presenting the results of each individual study in detail, we can better understand the impact of these factors on the study outcomes, providing a foundation for subsequent comprehensive and subgroup analyses. | 3.2  Results of Included Study Characteristics  3.3.1 Relationship between alcohol consumption and atherosclerosis |
| Results of syntheses | 20a | In this study, we conducted a comprehensive analysis of 26 studies involving 326,513 patients from 10 countries. The studies included 2 case-control studies, 10 cohort studies, and 14 cross-sectional studies. The types of alcohol consumption examined were primarily beer, spirits, and wine, while the sites of atherosclerosis varied, including the femoral artery, aorta, coronary artery, and carotid artery. The age range of participants was broad, with all being over 18 years old. Among the 26 studies, there were 215,986 male and 110,527 female patients. The risk of bias assessment revealed that most studies performed well in terms of data source clarity, inclusion and exclusion criteria, and control of confounding factors. However, some studies had shortcomings in describing quality assurance measures and handling missing data, which may introduce potential bias. | 3.2  Results of Included Study Characteristics  3.3.1 Relationship between alcohol consumption and atherosclerosis |
|  | 20b | We conducted a meta-analysis using a random-effects model to assess the relationship between alcohol consumption and atherosclerosis. The overall analysis showed no significant promoting effect of alcohol consumption on the development of atherosclerosis. However, subgroup analyses revealed several important trends. Alcohol consumption may increase the risk of atherosclerosis in specific countries (Japan, South Korea, Brazil, and Denmark), types of studies (cohort and case-control studies), arterial locations (coronary arteries), diagnostic criteria (clinical diagnosis and computed tomography) and gender(female). Additionally, varying levels of alcohol consumption appear to result in differing risks of the disease. | 3.3.1 Relationship between alcohol consumption and atherosclerosis |
|  | 20c | The heterogeneity test results showed significant variability among studies. To explore the sources of heterogeneity, we conducted subgroup analyses. The results indicated that the relationship between alcohol consumption and atherosclerosis varied across different countries, study types, arterial locations, and diagnostic criteria. For example, alcohol consumption significantly increased the risk of atherosclerosis in Japan and South Korea, but not in other countries. Moreover, the type and frequency of alcohol consumption may also influence the results. These findings suggest that the impact of alcohol consumption on atherosclerosis may be modulated by multiple factors, including environmental factors, lesion location, and individual characteristics. | 3.3.1 Relationship between alcohol consumption and atherosclerosis |
|  | 20d | To assess the robustness of the synthesized results, we conducted sensitivity analyses by sequentially excluding each study. The results remained consistent, indicating the reliability of our findings. Additionally, we used Egger's test to assess publication bias, and the results showed no significant publication bias. These analyses further support our conclusion that the relationship between alcohol consumption and atherosclerosis is not singular but is influenced by multiple factors. | 3.3.1 Relationship between alcohol consumption and atherosclerosis |
| Reporting biases | 21 | In this study, we assessed the risk of reporting bias using several methods. First, we employed Egger's test to quantify potential publication bias. The results indicated no significant publication bias among the included studies, suggesting that the published studies were relatively balanced and that unpublished negative results were unlikely to significantly impact the overall conclusions. Additionally, we used funnel plots to visually display the distribution of studies. Although the funnel plot showed slightly more studies on the left side than on the right, with some studies lying outside the 95% confidence interval, the overall distribution did not exhibit significant asymmetry, further supporting the conclusion of no significant publication bias. However, it is important to note that despite the lack of significant publication bias detected by Egger's test and funnel plot analysis, the presence of some heterogeneity among the included studies and the potential for unreported results or missing data in some studies cannot completely rule out the possibility of reporting bias. Future research should focus more on unpublished study results and reduce the impact of reporting bias through more comprehensive literature searches and data collection. | 3.3.1 Relationship between alcohol consumption and atherosclerosis |
| Certainty of evidence | 22 | In this study, we conducted a comprehensive evaluation of the quality of evidence from the included studies. Overall, the quality of evidence was influenced by several factors. First, the studies included exhibited heterogeneity in design, sample size, and quality, which may affect the consistency of the results. Second, publication bias may be present, as studies with positive or significant results are more likely to be published, potentially leading to an overestimation of the impact of alcohol. Additionally, most of the studies were observational and could not fully exclude the influence of confounding factors, thereby limiting the ability to infer causality. These limitations should be considered when interpreting the results and addressed in future research.  Nevertheless, through comprehensive analysis and sensitivity analysis, we concluded that the impact of these biases on the overall study results is limited, and the study results still have a certain level of reliability and reference value. Future research should further investigate the relationship between alcohol consumption and atherosclerosis, particularly the mechanisms and thresholds in different populations. | 3.3.1 Relationship between alcohol consumption and atherosclerosis |
| **DISCUSSION** | | |  |
| Discussion | 23a | Our study indicates that the impact of alcohol consumption on atherosclerosis is not singular but may interact with multiple factors, including environmental elements, lesion location, and individual characteristics. This finding aligns with existing literature, highlighting the complex relationship between alcohol consumption and cardiovascular health. Although our meta-analysis did not uncover overall evidence that alcohol significantly increases the risk of atherosclerosis, subgroup analyses revealed trends where alcohol might enhance risk under specific conditions. These results underscore the importance of considering individual differences when establishing health guidelines and preventive measures. | 5 Conclusions |
|  | 23b | The evidence base of this review has some limitations. Firstly, the included studies exhibit heterogeneity in design, sample size, and quality, which may affect the consistency of the results. Secondly, due to the presence of publication bias, only positive or significant studies may be published, potentially leading to an overestimation of alcohol's impact. Additionally, most studies are observational, which cannot entirely exclude the influence of confounding factors, limiting the ability to infer causality. These limitations should be considered when interpreting the results and should be addressed in future research. | 4 Discussion |
|  | 23c | The review process itself also has some limitations. For instance, our literature search may not have covered all relevant studies, especially those not indexed by major databases or published in non-English journals. Moreover, our analysis relies on data reported by study authors, which could be subject to reporting bias. In the process of data extraction and quality assessment, despite using a double-blind approach, subjectivity may still be present. Lastly, our meta-analysis failed to include all potential moderating variables, which might limit our comprehensive understanding of the relationship between alcohol consumption and atherosclerosis. | 4 Discussion |
|  | 23d | The findings of our study have significant implications for clinical practice, suggesting that doctors and public health experts should consider individual differences when providing advice on alcohol consumption. For policymakers, these findings emphasize the need to develop evidence-based guidelines to mitigate the potential negative impact of alcohol on cardiovascular health. For future research, our work points to the need for further investigation into the relationship between alcohol consumption and atherosclerosis, particularly the mechanisms and thresholds in different populations. Additionally, future studies should consider adopting stricter research designs, such as randomized controlled trials, to provide more robust causal evidence. | 4 Discussion |
| **OTHER INFORMATION** | | |  |
| Registration and protocol | 24a | To ensure methodological transparency and rigor, this meta-analysis followed the Meta-analysis of Observational Studies in Epidemiology (MOOSE) guidelines and the Preferred Reporting Items for Systematic Reviews and Meta-Analyses (PRISMA) statement. These guidelines provide a standardized framework for conducting and reporting systematic reviews and meta-analyses. Additionally, the study protocol has been registered with the International Platform of Registered Systematic Review and Meta-analysis Protocols (INPLASY) database (registration number: INPLASY202510031), further enhancing transparency and minimizing the risk of bias. | 2.6 Literature Screening and Data Extraction  2.1 Protocol and Registration |
|  | 24b | A protocol was not prepared. |  |
|  | 24c | We have not made any modifications to the information provided in the registration or protocol. |  |
| Support | 25 | This study was supported by the Jilin Provincial Administration of Chinese Medicine Science and Technology Program : Clinical Efficacy Study of Huotan Jiedu Huaban Tang in Intervening Atherosclerotic Plaques in High-Risk Coronary Heart Disease Population (Based on China-PAR Model) (Grant No. 20220203142SF). The funders played an important role in the process of data collation. | 9 Funding |
| Competing interests | 26 | The authors have no competing interests. | 7 Conflict of Interest |
| Availability of data, code and other materials | 27 | The following content is publicly available, and we will provide it in the supplementary files: template data collection forms; data extracted from included studies. | 11 Supplementary Material |

*From:*  Page MJ, McKenzie JE, Bossuyt PM, Boutron I, Hoffmann TC, Mulrow CD, et al. The PRISMA 2020 statement: an updated guideline for reporting systematic reviews. BMJ 2021;372:n71. doi: 10.1136/bmj.n71. This work is licensed under CC BY 4.0. To view a copy of this license, visit <https://creativecommons.org/licenses/by/4.0/>
